# Clustering-independent estimation of cell abundances in bulk tissues using single-cell RNA-seq data

**DOI:** 10.1101/2023.02.06.527318

**Published:** 2023-02-07

**Authors:** Rachael G. Aubin, Javier Montelongo, Robert Hu, Pablo G. Camara

**Affiliations:** Department of Genetics and Institute for Biomedical Informatics, Perelman School of Medicine, University of Pennsylvania, 3700 Hamilton Walk, Philadelphia, PA 19104.

**Keywords:** gene expression deconvolution, single-cell RNA-seq, ATAC-seq, spatial transcriptomics, ependymoma, glioma

## Abstract

Single-cell RNA-sequencing has transformed the study of biological tissues by enabling transcriptomic characterizations of their constituent cell states. Computational methods for gene expression deconvolution use this information to infer the cell composition of related tissues profiled at the bulk level. However, current deconvolution methods are restricted to discrete cell types and have limited power to make inferences about continuous cellular processes like cell differentiation or immune cell activation. We present ConDecon, a clustering-independent method for inferring the likelihood for each cell in a single-cell dataset to be present in a bulk tissue. ConDecon represents an improvement in functionality and accuracy with respect to current deconvolution methods. Using ConDecon, we discover the implication of neurodegenerative microglial inflammatory pathways in the mesenchymal transformation of ependymoma, recapitulate spatial patterns of cell differentiation during zebrafish embryogenesis, and make temporal inferences from bulk ATAC-seq data. Overall, ConDecon significantly enhances our understanding of dynamic cellular processes within bulk tissue samples.

## Introduction

Biological tissues are complex systems composed of millions of cells interacting to produce biological function. Characterizing the cellular composition and heterogeneity of tissues is thus fundamental to understanding the relation between cellular phenotypes and tissue function, and it has been a major area of investigation for over a century^1–3^. Advances in high-throughput single-cell RNA sequencing (RNA-seq) have revolutionized the study of tissue composition by enabling the transcriptomic characterization of cell types and states without the need for pre-defined markers^4,5^. However, establishing robust associations between tissue cell composition and other data, such as clinical data, requires generating, profiling, and analyzing large cohorts of samples, which is often technically, computationally, and financially prohibitive by single-cell RNA-seq. In addition, the tissue dissociation and cell encapsulation techniques involved in single-cell RNA-seq can lead to the underrepresentation of some cell populations^6^. Since transcriptomic profiling of tissues at the bulk level does not suffer from these limitations, an enticing alternative is to use the bulk-level gene expression profile of each sample to computationally infer the abundance of each cell population in the sample^7,8^. This approach is known as gene expression deconvolution.

**Figure 1.**
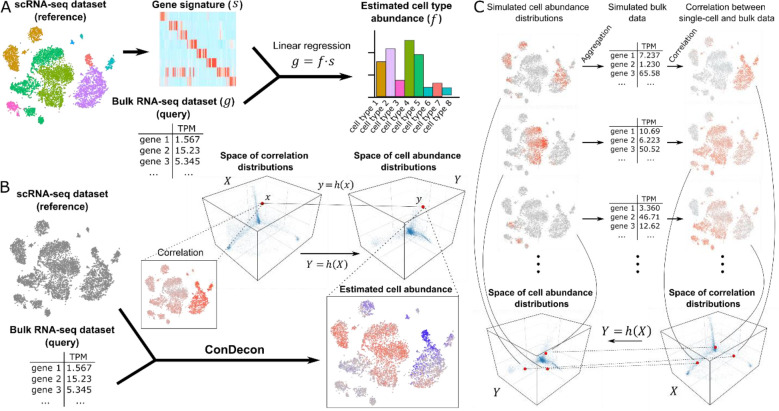
A clustering-independent approach for cell abundance inference from gene expression data of bulk tissues. **(A)** Conventional methods for gene expression deconvolution of bulk tissues cluster a reference single-cell RNA-seq dataset into discrete cell populations and perform differential gene expression to build a gene expression signature matrix for the discrete cell populations. The problem of estimating discrete cell type abundances is then formulated as a linear regression problem. **(B)** The approach of ConDecon to gene expression deconvolution differs substantially from that of conventional methods. It takes as input a bulk RNA-seq query dataset and a reference single-cell RNA-seq dataset. It then computes the rank correlation between the gene expression profiles of the bulk RNA-seq dataset and each cell in the single-cell dataset using the most variable genes. The resulting correlations are represented by a point in the space of possible correlation distributions with support on the single-cell RNA-seq latent space. ConDecon then maps that point into a point in the space of possible cell abundance distributions with support on the single-cell RNA-seq latent space. **(C)** The model of ConDecon is trained by simulating multiple cell abundance distributions with support on the single-cell RNA-seq latent space by means of a Gaussian mixture model. For each simulated distribution, a synthetic bulk RNA-seq dataset is constructed by aggregating the gene counts, and the rank correlation between the gene expression profiles of the synthetic bulk RNA-seq dataset and each cell in the single-cell dataset is computed using the most variable genes. The paired cell abundance and correlation distributions are then used to learn the function *h* that maps the spaces of possible correlation distributions and cell abundance distributions with support on the single-cell RNA-seq latent space.

Current methods for gene expression deconvolution use matrix regression, such as support vector^9,10^, least-squares^11–15^, elastic net^16^, or least absolute deviation regression^17^, to represent the overall gene expression profile of the tissue as a linear combination of cell type-specific gene expression signatures (Figure 1A). These gene expression signatures are directly provided by the user or built by the method from a reference single-cell RNA-seq dataset by clustering and differential gene expression analysis of the single-cell transcriptomes. These approaches work particularly well when the cell types present in the sample have very distinct gene expression profiles and form discrete clusters in the single-cell gene expression space^18,19^. However, poorly characterized cell states or continuous cellular processes, such as cell differentiation or immune cell activation, cannot be accurately described in terms of discrete cell populations and often involve colinear gene expression signatures. These nuances are usually lost inside broader populations, limiting the resolution to detect small changes in cell state. In addition, by averaging transcriptomic variability within cell clusters, the output of these methods strongly depends on the choice of clustering algorithm and parameters. Consistent with these limitations, two recent studies found that the specificity of the reference gene expression signatures that are used is the greatest determinant of accuracy in current methods for gene expression deconvolution^19,20^. Therefore, there is a need for clustering-independent approaches that can take full advantage of reference single-cell RNA-seq data to infer cell abundances in bulk tissue samples with high phenotypic resolution.

Here we present a deconvolution method, named ConDecon, for inferring cell abundances from gene expression data of bulk tissues without relying on cluster labels or cell-type specific gene expression signatures at any step. The aim of ConDecon is to infer a probability distribution across a reference single-cell RNA-seq dataset that represents the likelihood for each cell in the reference dataset to be present in the query bulk tissue. By means of multiple analyses of simulated and real data from well-characterized systems, we demonstrate that ConDecon can be used to accurately estimate cell abundances in bulk tissues composed of discrete cell types and continuous cellular processes, where the application of current deconvolution methods is limited. The estimates that result from aggregating ConDecon’s cell probabilities across cells of the same type are highly concordant with flow cytometry measurements, and often more accurate than those from current clustering-based deconvolution methods.

We demonstrate the utility of ConDecon with three applications. First, by using it with single-cell and bulk gene expression data of pediatric ependymal tumors^21^, we uncover continuous changes in tumor infiltrating microglia related to the mesenchymal transformation of tumor stem cells. These results, which we confirm by immunohistochemistry, show that microglia surrounding mesenchymal tumor regions acquire an activation phenotype similar to that of microglia in neurodegenerative disease lessions^22–24^. Second, we use ConDecon to deconvolve the gene expression profile of individual spots in spatial transcriptomic data of the zebrafish embryo^25^. Using these results, we transfer continuous cell differentiation trajectories from the reference single-cell RNA-seq data into the spatial data and infer spatial patterns of cell differentiation during embryogenesis. Last, we build upon the generality of the approach implemented in ConDecon to deconvolve other omics data modalities and estimate cell abundances in bulk ATAC-seq data using reference single-cell ATAC-seq data. Altogether, these applications demonstrate that ConDecon enables previously elusive analyses of dynamic cellular processes in bulk tissues and represents an increase in functionality and phenotypic resolution with respect to current methods for gene expression deconvolution.

## Results

### Clustering-independent cell abundance estimation from gene expression data of bulk tissues

To overcome the inherent limitations of cell-type specific gene expression signatures in the deconvolution of gene expression data from bulk tissues, we developed ConDecon, a clustering-independent method for inferring changes in cell abundance based on reference single-cell RNA-seq data provided by the user. ConDecon uses the gene expression count matrix and latent space of the reference single-cell RNA-seq dataset to estimate the likelihood for each cell in the dataset to be present in the query bulk tissue sample (Figure 1B). For that purpose, it assumes that the similarity between the gene expression profile of cells in the single-cell dataset and that of the bulk tissue sample, as measured by their rank correlation, can be used as a proxy for inferring this likelihood function (Figure 1B). The goal of ConDecon is thus to learn a map *h*(*X*): *X* → *Y* between the space *X* of possible rank correlation distributions and the space *Y* of possible probability distributions on the single-cell gene expression latent space. ConDecon introduces coordinates in *X* and *Y* by expanding the distributions in a basis, such as principal components or diffusion maps, and represents *h*(*X*) as a polynomial function on the coordinates.

To learn *h*(*X*), ConDecon simulates bulk transcriptomic data by aggregating the gene expression profiles of cells sampled from the single-cell reference dataset according to a randomly generated mixture of Gaussian distributions with variable number of components (Figure 1C). Each simulated probability distribution represents a point *y* ∈ *Y*. The correlation coefficient between the gene expression profiles of the simulated bulk dataset and each of the cells in the single-cell dataset then provides a point *x* ∈ *X* such that *y* = *h*(*x*) (Figure 1C). By using this procedure to simulate many bulk datasets, it is possible to fit the model for *h*(*X*). With the fitted model, ConDecon can then infer the distribution of cell abundances for any query bulk sample of the same tissue type as the single-cell reference dataset. This clustering-independent approach is therefore conceptually different from the regression-based approach used by current methods for gene expression deconvolution and takes full advantage of all the variability contained in the reference single-cell dataset.

### Estimation of cell abundances in simulated RNA-seq data of discrete cell types and continuous cellular processes

To demonstrate the ability of ConDecon to infer changes in cell state, we simulated single-cell RNA-seq data from a broad range of configurations using the algorithm Splatter^26^. Splatter uses a gamma-Poisson model to simulate gene-by-cell RNA count matrices of complex tissues. From each simulated single-cell dataset, we derived several query bulk RNA-seq datasets by non-uniformly sampling cells from the single-cell dataset and aggregating their gene expression profile. We then used ConDecon to estimate cell abundances in each simulated bulk dataset and compared them to the ground-truth abundances.

**Figure 2.**
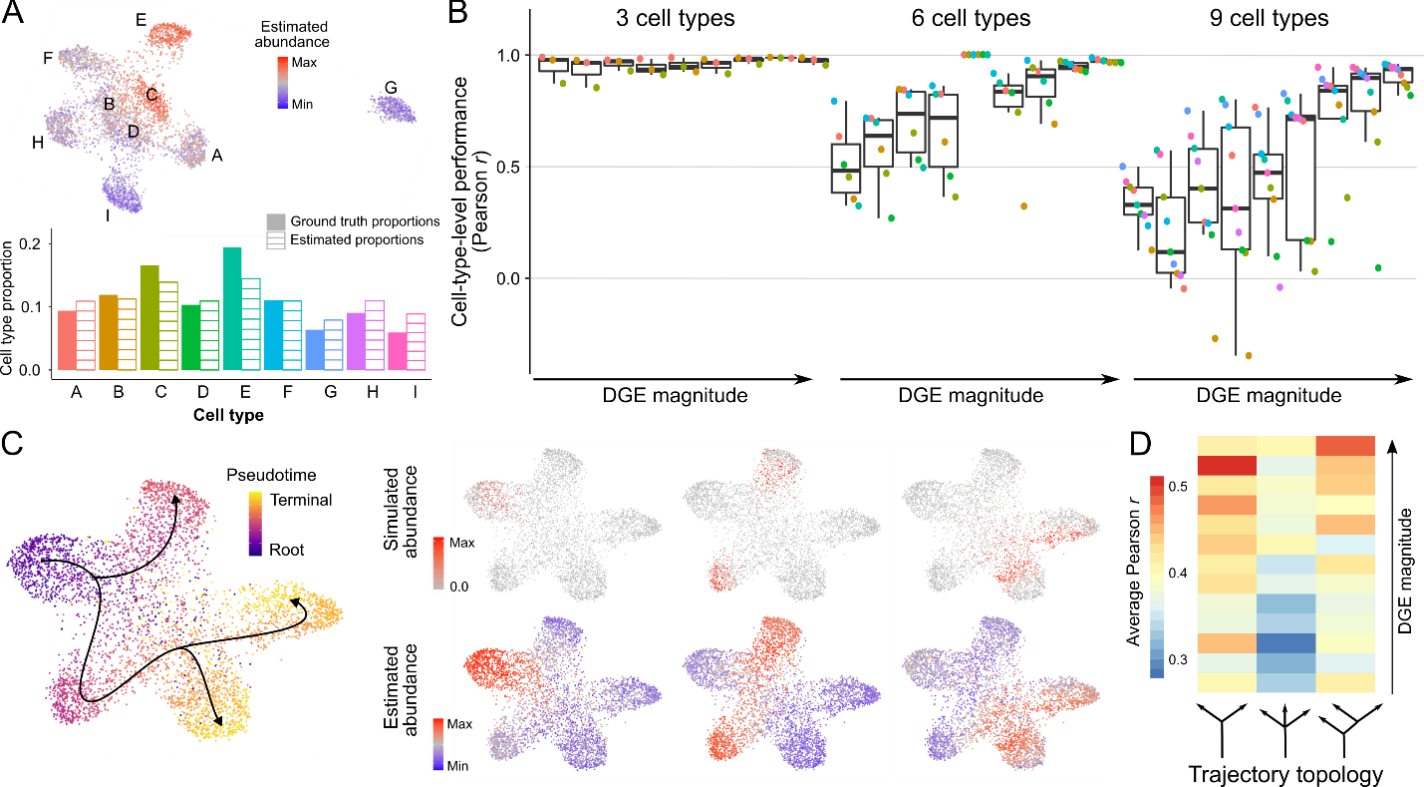
Estimation of cell abundances from simulated gene expression data of discrete cell populations and continuous cellular processes. **(A)** Estimation of cell population abundances in a simulated bulk RNA-seq dataset with 9 discrete cell populations (named A to I). Single-cell RNA-seq data was simulated using Splatter and the gene expression profiles of individual cells sampled with different probability from each cell population were pooled to construct a synthetic bulk RNA-seq dataset. The UMAP representation of the single-cell RNA-seq data is colored by ConDecon’s estimated cell abundances (top). The aggregated cell abundance estimates across each cell population recapitulate the simulated cell population abundances (bottom). **(B)** Pearson correlation coefficient between the simulated and estimated abundances for each cell population in 675 simulated bulk RNA-seq datasets with 3, 6, or 9 discrete cell populations and varying degree of differential gene expression (DGE). **(C)** Cell abundance estimation in 3 simulated bulk RNA-seq datasets of a continuous cell differentiation process with 1 precursor and 3 terminally differentiated cell states. The UMAP representation of the simulated single-cell RNA-seq data is colored by the simulated pseudotime (left). The cell abundances estimated with ConDecon in each of the simulated bulk RNA-seq datasets (right, bottom) recapitulate the simulated abundances that were used to construct the bulk data (right, top). **(D)** Average Pearson correlation coefficient between the simulated and estimated cell abundances in 975 simulated bulk RNA-seq datasets with 3 topologies for the cell differentiation trajectories and varying degree of differential gene expression (DGE). See also Figures S1 and S2.

We first tested ConDecon in simple scenarios where the bulk tissue consists of a set of discrete cell populations. In these simulations, each cell population was taken to be homogeneous up to some random variability. We generated 27 single-cell RNA-seq datasets consisting of 3, 6, or 9 cell types and varying degrees of differential gene expression. From each dataset, we derived 25 bulk RNA-seq datasets where we varied the sampling probability of each cell type to simulate different cell type abundances. To quantify the concordance between the estimated and simulated cell abundances, we aggregated the estimates of ConDecon across cells of the same type. The estimated and simulated abundances for each cell type were strongly correlated in most cases (Figures 2A, 2B, and S1A, average Pearson’s *r* = 0.75, average *p*-value = 0.06). This correlation was higher for samples with fewer cell types or higher differential gene expression (Figure 2B). Thus, ConDecon can be efficiently used to deconvolve gene expression in bulk tissues consisting of discrete cell populations, where standard methods for gene expression deconvolution can be also applied.

We next simulated single-cell RNA-seq data from continuous cellular processes, such as cell differentiation, for which the application of conventional gene expression deconvolution methods is contrived. We generated 39 single-cell RNA-seq datasets consisting of cell differentiation trajectories with three different topologies and varying differential gene expression levels. Each trajectory consisted of a precursor and two or three terminally differentiated cell states (Figure 2B). For each simulated single-cell RNA-seq dataset, we generated 25 bulk datasets by sampling cells based on a random Gaussian kernel on pseudotime (Figure 2C). The single-cell abundance estimates of ConDecon were again strongly correlated with the ground-truth abundances (Figures 2C, 2D, and S2A, average Pearson’s *r* = 0.40, average *p*-value = 2 × 10^−9^) and their accuracy improved with the amount of differential gene expression (Figure 2D).

To evaluate the stability of these results, we repeated these analyses using different choices for the parameters of ConDecon, including the dimensionality of the spaces *X* and *Y* and the number of variable genes used in the computation of the rank correlation. This analysis showed that the estimates of ConDecon are stable against different choices for its parameters, only observing a substantial decrease in the accuracy of the estimates for small values of the parameters (less than 500 genes and 5 dimensions) (Figures S1B, S1C, and S2B). Taken together, the application of ConDecon to simulated data demonstrates the validity of its clustering-independent approach to deconvolve gene expression data from complex tissues consisting of both discrete cell populations and continuous cellular processes.

### ConDecon’s estimates of cell type abundances are comparable or superior to those from clustering-based methods

**Figure 3.**
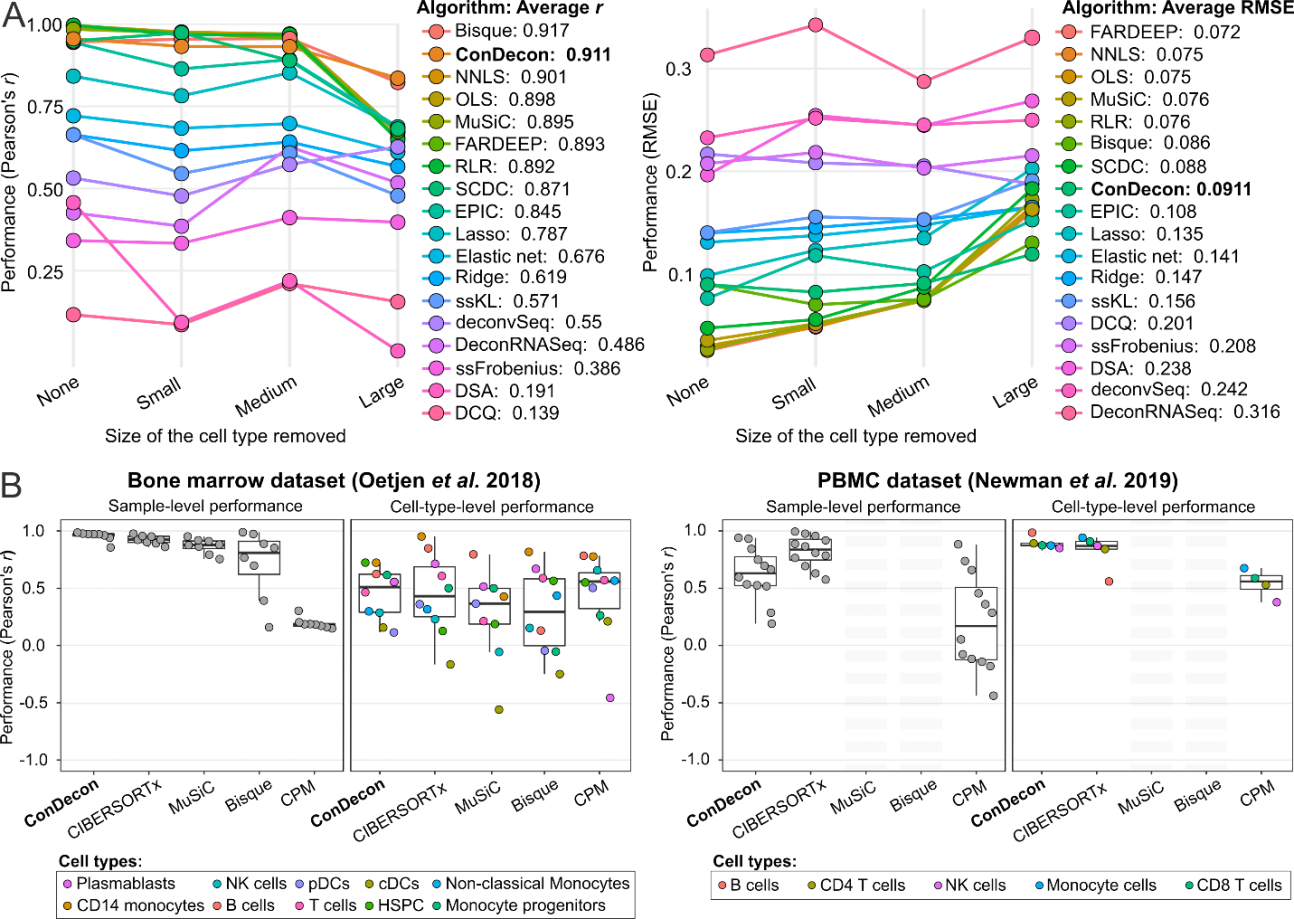
Benchmarking the cell type abundance estimates of ConDecon in comparison to current gene expression deconvolution methods. **(A)** The cell abundance estimates of ConDecon were aggregated into cell type abundance estimates and compared with those of 17 other deconvolution methods across 6 datasets using the benchmarking pipeline of Avila-Cobos *et al.*^27^. For each algorithm, the Pearson’s correlation coefficient (left) and the root mean squared error (RMSE) (right) of the estimates, combined across samples and cell types, is shown for cases where there is none, one small, one medium, or one large cell population missing in the reference single-cell data. **(B)** Comparison between cell type abundance estimates derived from FACS data and those from ConDecon and 4 other deconvolution methods that were specifically devised to use single-cell RNA-seq data as reference data. Two bulk RNA-seq datasets consisting of 8 bone marrow^30^ (left) and 12 PBMC^10^ (right) samples, for which paired FACS data are available, were considered for this evaluation. The sample-level and cell-type-level performance are shown for each algorithm in each dataset. We were unable to apply MuSiC and Bisque to the PBMC dataset since these methods require that the reference single-cell RNA-seq data consists of at least 2 biological replicates. See also Figures S3 and S4.

We used a published benchmarking pipeline^27^ to perform a systematic comparison of the cell type abundance estimates of ConDecon with those produced by 17 other methods for gene expression deconvolution. The pipeline uses single-cell RNA-seq data to simulate bulk RNA-seq datasets of mixtures of discrete cell types. It then evaluates the accuracy and stability of the estimates produced by each algorithm when none or one cell type in the query sample is missing in the reference data^27^. For these comparisons, we used six single-cell RNA-seq datasets of peripheral blood mononuclear cells (PBMC)^10^, pancreas^11,28,29^, bone marrow^30^, and kidney^31^. We assessed each algorithm based on the Pearson’s correlation coefficient and the root mean squared error (RMSE) of the estimates combined across samples and cell types^27^. In this comparative study, ConDecon performed similarly to the best performing clustering-based method, outperforming 16 of the 17 other methods based on the average correlation (Figures 3A and S3, average combined Pearson’s *r* = 0.91, average *p*-value < 10^−15^). The average RMSE of ConDecon was also similar to that of current methods for gene expression deconvolution (Figures 3A and S3, average combined RMSE = 0.091). Expectedly, the accuracy of the estimates produced by all the algorithms decreased with the size of the missing population in the reference single-cell data. However, the estimates of ConDecon and Bisque^32^ were more robust against missing large cell populations (Figures 3A and S3).

A caveat of the benchmarking pipeline is that bulk RNA-seq data are simulated from single-cell RNA-seq data and may therefore lack some of the technical features present in actual bulk RNA-seq datasets. To overcome this limitation, we also considered two published bulk RNA-seq datasets consisting of 8 bone marrow^30^ and 12 PBMC^10^ samples for which paired fluorescence-activated cell sorting (FACS) data are also available. We compared the estimates of ConDecon in these bulk RNA-seq datasets to those of CIBERSORTx^10^, MuSiC^12^, Bisque^32^, and CPM^33^, and to the cell type abundances inferred by FACS. Like ConDecon, these methods for gene expression deconvolution have been specifically devised to use single-cell RNA-seq data as reference. Specifically, CIBERSORTx accounts for platform-specific variation when comparing single-cell and bulk gene expression levels, whereas MuSiC and Bisque leverage multi-subject single-cell expression data to improve the accuracy of the estimates. These three algorithms seek to infer the abundance of each cell type in the query sample, whereas CPM aims to reconstruct the continuous spectrum of cell states within single query cell type. For that purpose, CPM partitions the gene expression space of the cell type into smaller discrete domains and uses a bootstrapped support vector regression approach to infer the abundance of each domain^33^. However, CPM is not designed to be used with tissues that comprise multiple cell types.

In our comparison with the estimates from FACS data, we observed that ConDecon outperformed the other methods based on the average Pearson’s correlation across samples (cell-type-level performance) (Figure 3B). In addition, it showed the highest average correlation across cell types (sample-level performance) in the deconvolution of bone marrow data. In contrast, CIBERSORTx had a better sample-level performance in the deconvolution of PBMC data (Figure 3B). This was in part expected, since CIBERSORTx was originally benchmarked and optimized using this dataset^10^. We were unable to apply MuSiC and Bisque to the PBMC dataset since these methods require that the reference single-cell RNA-seq data consists of at least 2 replicates. In addition, CPM did not perform as well as the other methods in these analyses due to the presence of heterogeneous mixtures of cell types^33^.

Varying the parameters of ConDecon, we found that a minimum of 500 variables genes and 5 dimensions was needed for the cell type abundance estimates to be in good agreement with the FACS data (Figure S4), consistent with the results of our simulations.

Altogether, these analyses show that aggregating the single-cell abundance estimates of ConDecon into discrete cell type abundances leads to comparable or better estimates than those from widely used clustering-based deconvolution methods.

### Inference of continuous changes in B-cell maturation with ConDecon

**Figure 4.**
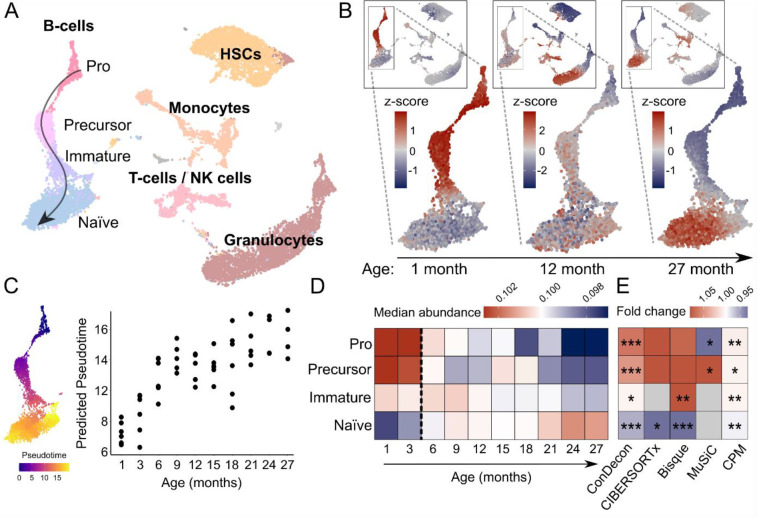
Identification of age-associated changes in B-cell maturation using bulk bone marrow tissues. **(A)** UMAP representation of the single-cell RNA-seq data of 14,107 cells from the bone marrow of 13 mice with ages between 1 and 27 months profiled by the Tabula Muris Consortium^34^. The representation is labelled by the major annotated cell populations. The developmental lineage of B-cells has been subclustered into Pro B-cells, precursor B-cells, immature B-cells, and mature naïve B-cells. **(B)** Single-cell abundances inferred by ConDecon for three bone marrow samples from 1-, 12-, and 27-months old mice profiled with bulk RNA-seq. The youngest mouse has a high abundance of Pro and precursor B-cells, whereas the oldest mouse has a high abundance of mature B-cells (Pearson’s *r* = 0.77, *p*-value = 2 × 10^−11^) **(C)** Average pseudotime inferred by ConDecon for the B cells in each bulk sample as a function of the mice age, for bone marrow samples of 53 mice profiled with bulk RNA-seq. As expected, the inferred average pseudotime increases with the age of mice. For reference, the UMAP representation of the B-cell lineage colored by the pseudotime is also shown. **(D)** Median aggregated cell abundances inferred by ConDecon for each B-cell subpopulation as a function of the age of mice. **(E)** Fold change in the median aggregated cell abundances between young (≤ 3 months) and adult (> 3 months) mice according to the estimates of ConDecon and four other algorithms specifically devised to use single-cell data as reference. Clustering-based methods (CIBERSORTx, Bisque, and MuSiC) had limited power to capture differences between young and adult mice, whereas CPM inferred very small changes in abundance. (2-sided Wilcoxon rank sum test; *: *p*-value ≤ 0.05, **: *p*-value ≤ 0.01, ***: *p*-value ≤ 0.001).

Having tested ConDecon with tissues that consist of mixtures of relatively homogeneous cell types, we next used it to study changes in single-cell abundance associated with continuous cellular processes. We considered single-cell and bulk RNA-seq data of bone marrow from mice with ages between 1 and 27 months^34^ and used these data to study changes in cell abundance associated with development and aging. We used the well-characterized changes in B-cell abundance that occur during postnatal development^35^ as a test system. Using an integrated representation of these single-cell data with no age labels as reference, ConDecon was able to recapitulate from the bulk data the continuous transition from an abundance of pro B-cells in young mice (≤ 3 months) to an abundance of naïve mature B-cells in fully developed mice (Figures 4A–D, Pearson’s correlation between the age of mice and the average inferred pseudotime of the B-cells in each mouse *r* = 0.77, *p*-value = 2 × 10^−11^), in agreement with previous results based on FACS data^35^. Compared to the alternative approach of sub-clustering the continuous B-cell trajectory into discrete cell subpopulations and using conventional deconvolution methods to infer the abundance of each subpopulation, ConDecon showed a higher power to identify changes in cell abundance (Figure 4E), possibly due to the fact that conventional methods do not account for intra-cluster variability in cell state abundance. For instance, although the overall immature B-cell population was not enriched in 1 month-old mice, a subset of these cells with a gene expression profile close to that of precursor B-cells was already present at this age (Figure 4B). Thus, ConDecon’s inferences are not restricted to discrete cell populations and can be used to infer changes in cell abundance along continuous cellular trajectories with high resolution.

### Microglia acquire a neurodegenerative associated gene expression phenotype during the mesenchymal transformation of pediatric ependymoma

**Figure 5.**
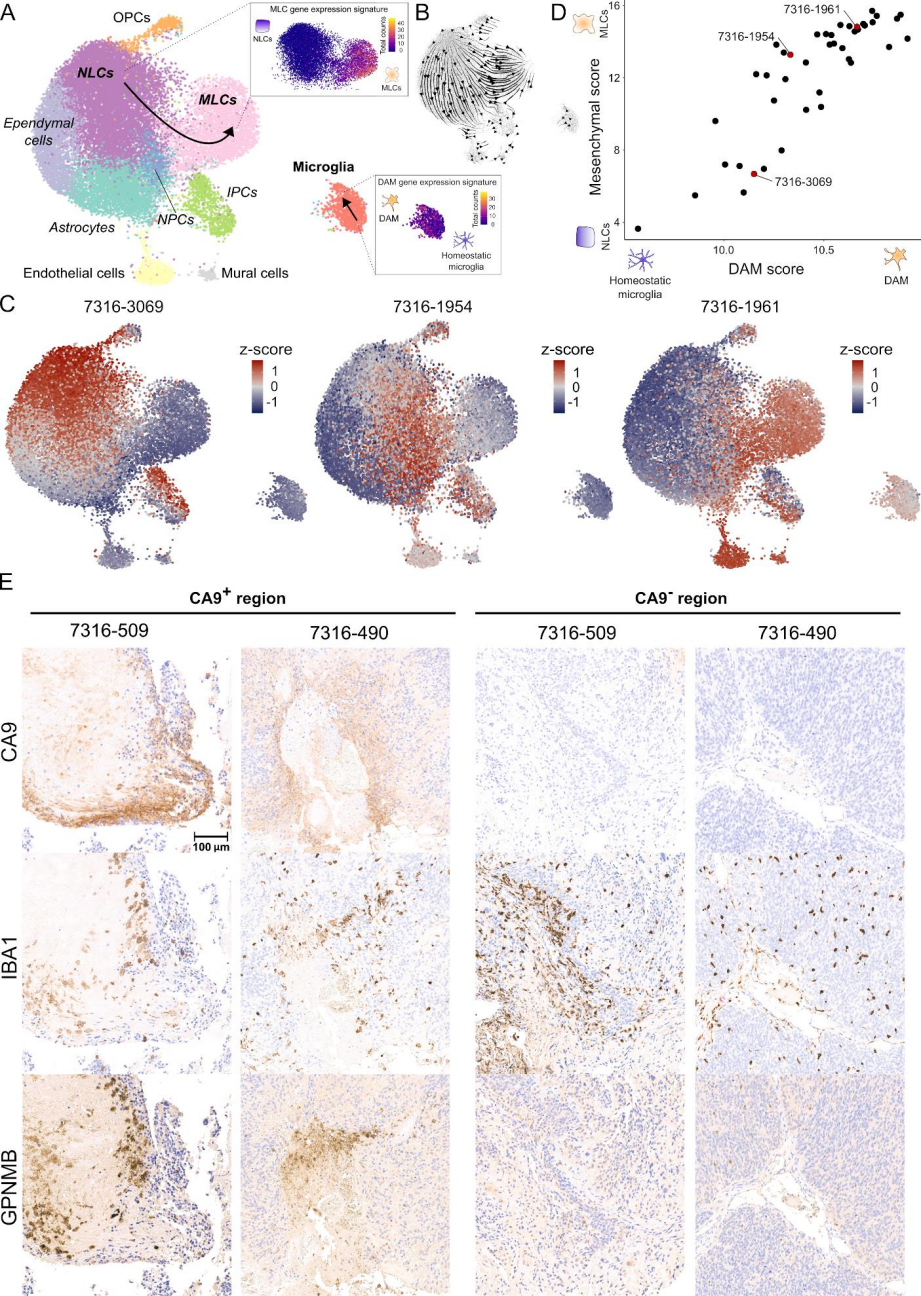
Tumor infiltrating microglia express neurodegenerative disease signatures during the mesenchymal transformation of pediatric ependymoma. **(A)** UMAP representation of 25,349 cells from 9 posterior fossa ependymal tumors profiled with single-nucleus RNA-seq in Aubin *et al.*^21^. The representation is colored by the annotated cell populations. NLCs: neuroepithelial tumor stem cells; MLCs: mesenchymal tumor cells; NPCs: neural progenitor tumor cells; IPCs: intermediate progenitor tumor cells. The two studied transitions, corresponding to the transformation of NLCs into MLCs and the acquisition of a disease-associated microglia (DAM) phenotype by tumor-infiltrating microglia are schematically indicated. In the inserts, the UMAP representation is colored by the total number of counts of genes belonging to the MLC and DAM gene expression signatures. **(B)** The RNA velocity stream plot showing consistency with the two transitions is shown for reference. **(C)** Single-cell abundance estimates computed with ConDecon for three posterior fossa pediatric ependymal tumors profiled with bulk RNA-seq that span the entire neuroepithelial-to-mesenchymal-like transition. Tumor 7316–3069 has a high abundance of NLCs and small abundance of infiltrating microglia. Most of the microglia are in a homeostatic transcriptional state. In contrast, tumor 7316–1961 has a high abundance of MLCs and infiltrating microglia, and most microglia are in a DAM state. Tumor 7316–490 represents an intermediate state. **(D)** DAM and mesenchymal pseudotimes inferred by ConDecon for 42 posterior fossa pediatric ependymal tumors profiled with RNA-seq at the bulk level. For each tumor, DAM and mesenchymal scores are defined respectively by the average total number of counts of the DAM or MLC gene expression signature inferred by ConDecon for the microglia and tumor cells in each bulk sample. The two scores are correlated (Pearson’s *r* = 0.86, *p*-value < 4 × 10^−13^), indicating that the transition of NLCs into MLCs in the tumor is strongly associated with the transition of infiltrating microglia from a homeostatic transcriptional state onto a DAM state. **(E)** Immunohistochemistry staining of adjacent tissue sections from two pediatric ependymal tumors (7316–509 and 7316–490). Each tumor was stained for CA9 (a marker of mesenchymal regions), IBA1 (a microglial marker), and GPNMB (a DAM marker). In both tumors, microglia that are infiltrating CA9^+^ mesenchymal regions express GPNMB, whereas microglia in neuroepithelial regions of the tumor do not have detectable levels of GPNMB. Scale bar: 100 μm. See also Figures S5 and S6.

Pediatric ependymoma is a brain cancer that is particularly aggressive in young children due to its very relapsing pattern and lack of effective chemotherapies^36–38^. Recent single-cell RNA-seq studies of ependymal tumors have identified a subpopulation of tumor cells with a mesenchymal-like gene expression profile that is associated with abundant microglia infiltration and poor prognosis^21,39–41^. Mesenchymal-like tumor cells in ependymoma are thought to derive from neuroepithelial-like tumor cells by activation of brain injury repair and neuroinflammation pathways in response to microglia-secreted cytokines^21,41^. To investigate this process, we used ConDecon to study the changes in the gene expression profile of tumor-infiltrating microglia during the mesenchymal transformation of tumor cells. We considered a cohort of 42 ependymal tumors profiled with RNA-seq at the bulk-level and a reference single-nucleus RNA-seq atlas of primary and metastatic ependymoma^21^. Our analysis revealed that the abundance of mesenchymal-like tumor cells and microglia in each sample are positively correlated (Pearson’s *r* = 0.70, *p*-value = 3 × 10^−7^), in agreement with previous results using clustering-based deconvolution methods^21^. However, it also showed that tumor infiltrating microglia describe a previously unnoticed trajectory in the gene expression space across samples (Figure S5), which suggests a change in the expression profile of these cells during the mesenchymal transformation of ependymoma. Differentially expressed genes at one end of this trajectory included genes that are characteristic of neurodegenerative disease-associated microglia (DAM)^22–24^, such as *Apoe*, *Trem2*, *Gpnmb*, *Csf1*, *Spp1*, and *Il1b*. Using the DAM and mesenchymal gene expression signatures, we introduced a pseudotime in each of the two trajectories describing the transition of neuroepithelial-like tumor cells into mesenchymal-like tumor cells and homeostatic microglia into DAM (Figure 5A). The ordering of cells along these trajectories was consistent with the RNA velocity vector field^42,43^ (Figure 5B). By computing the expected pseudotime for the microglia and mesenchymal-like tumor cells of each bulk sample based on the probabilities inferred by ConDecon, we found that the state of each sample along the microglial trajectory was strongly correlated with its state along the epithelial-to-mesenchymal-like transition (Figures 5C, 5D, and S5, Pearson’s correlation coefficient between average pseudotime in each trajectory *r* = 0.86, *p*-value < 4 × 10^−13^). Thus, as tumor cells gradually progress from a neuroepithelial-like state onto a mesenchymal-like state, tumor infiltrating microglia express a DAM gene expression signature consisting of genes involved in phagocytosis and neuroinflammation. To validate these results in patients, we performed immunohistochemistry of two primary and one metastatic posterior fossa A ependymal tumors. We stained adjacent tissue sections for CA9, which is expressed by mesenchymal-like ependymoma tumor cells^39,41^, IBA1, which is expressed by microglia, and GPNMB, which is expressed by DAM^22^ and is involved in the pathogenesis of Parkinson’s disease^44^. Consistent with the predictions of ConDecon, the immunohistochemistry data showed that microglia surrounding or infiltrating mesenchymal regions of the tumors expressed high levels of GPNMB (Figures 5E and S6). In contrast, we did not detect the expression of GPNMB in microglia infiltrating non-mesenchymal regions of the tumors (Figures 5E and S6). Altogether, these results suggest that some of the key microglial inflammatory pathways implicated in neurodegenerative disorders are also involved in the mesenchymal transformation of pediatric ependymoma.

### Clustering-independent estimation of cell abundances using other omics data modalities

**Figure 6.**
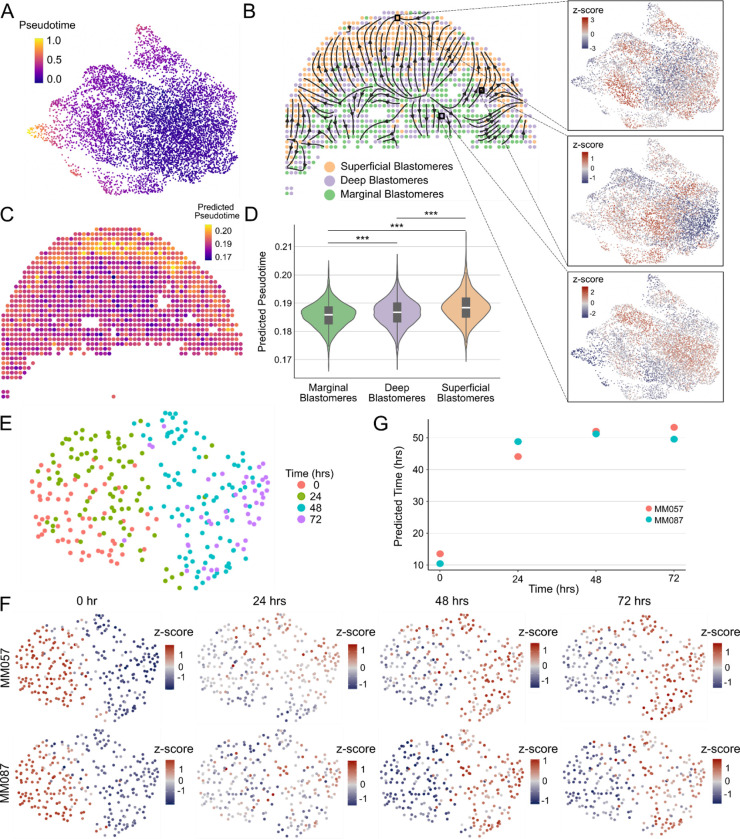
Deconvolution of spatial transcriptomics and ATAC-seq data using ConDecon. **(A)** UMAP representation of the single-cell RNA-seq data of 7,424 blastomere cells from 3.3 hpf zebrafish embryos^25^. The representation is colored by pseudotime associated with the maturation of blastomere cells. **(B)** Spatial representation of a 3.3 hpf zebrafish embryo tissue section profiled with Stereo-seq^25^. Each pixel is labelled according to its major abundance of marginal, deep, or superficial blastomeres. The spatial cell differentiation trajectories inferred with ConDecon are overlaid on the representation. Differentiation proceeds from marginal blastomeres to deep and superficial blastomeres. The UMAP representation of the reference single-cell dataset colored by the cell abundances estimated with ConDecon is also shown for 3 representative Stereo-seq pixels. **(C)** The same tissue section as in (A) is colored by the average pseudotime of the cells in each pixel estimated with ConDecon. **(D)** Violin plot showing the distribution of estimated pseudotimes for pixels classified as marginal, deep, or superficial across 10 tissue sections profiled with Stereo-seq. Boxes represent the median and interquartile range. Wilcoxon rank-sum test. ***: p-value < 10^−15^. **(E)** UMAP representation of the single-cell ATAC-seq data of a patient-derived melanoma cell line (MM087) profiled 0, 24, 48, and 72 hours after knocking out SOX10^53^. **(F)** The same representation as in (E) is colored by the estimated single-cell abundances for 8 samples from 2 melanoma cell lines (MM057 and MM058) profiled with bulk ATAC-seq 0, 24, 48, and 72 hours after knocking out SOX10. **(G)** Average sampling time estimated with ConDecon for the cells in each of the 8 bulk ATAC-seq samples as a function of the actual sampling time. As expected, the inferred average sampling time for the cells increases with the actual sampling time of the bulk sample (Pearson’s *r* = 0.83, *p*-value = 0.01) See also Figure S7.

The general approach of ConDecon for estimating cell abundances can be applied to other omics data modalities such as spatial transcriptomics or chromatin accessibility data. To evaluate the utility of using ConDecon to deconvolve spot-based spatial transcriptomic data using single-cell RNA-seq data as reference, we considered published Stereo-seq data of 10 zebrafish embryos profiled 3.3 hours post-fertilization (hpf)^25^. At this stage of development, the embryo consists of ~4,000 blastomere cells arranged in >11 tiers with varying levels of cell differentiation. We used ConDecon to infer the distribution of cell abundances across each tissue section, where each pixel was treated as a bulk sample. Since pixel size in the processed Stereo-seq data is approximately 10 μm^25^, each pixel is expected to receive contributions from 1 to 3 cells. As a reference dataset, we considered single-cell RNA-seq data of 3.3 hpf embryos from the same study and used diffusion pseudotime^45^ to parameterize the differentiation of blastomere cells in these data (Figures 6A and S7). We then used the cell probabilities inferred by ConDecon for each pixel to deconvolve pseudotime and derive trajectories of cell differentiation in the spatial data (Figures 6B, 6C, and S7). The resulting trajectories recapitulated the known spatial patterns of cell differentiation in the blastodisc, where the differentiation sequence progresses from marginal blastomere cells into deep and superficial blastomere cells^46^ (Figure 6D). Compared to current methods for deconvolving spot-based transcriptomic data^47–51^, with the exception of DestVI^52^ which can perform continuous estimates of cell state within cell clusters, the clustering-independent approach of ConDecon can be used to deconvolve continuous features like cell differentiation pseudotime and study the relation between cell differentiation and tissue architecture.

We next evaluated the ability of ConDecon to estimate cell abundances from bulk ATAC-seq data using single-cell ATAC-seq data as reference. We considered published bulk and single-cell ATAC-seq data of two short-term cultures derived from melanoma patient biopsies^53^. In these cultures, the transcription factor SOX10 was knocked down by siRNA and cells were sampled at 0, 24, 48, and 72 hours after SOX10 knockdown. To assess the performance of ConDecon in deconvolving bulk ATAC-seq data, we combined the single-cell ATAC-seq data from different time points into a single reference single-cell dataset and compared the sampling time of the cells inferred by ConDecon for each of 8 bulk samples with the actual sampling time of the samples (Figures 6E and 6F). To maximize variability between the reference and query datasets and improve the quality of the reference dataset, we only considered one of the two cell lines in this dataset (Figure 6E). In these analyses, ConDecon inferred a higher abundance of reference cells from the same sampling time than the query bulk sample, independently of the specific cell line of the query sample (Figures 6F and 6G, Pearson’s correlation coefficient between ConDecon’s estimated sampling time and actual sampling time *r* = 0.83, *p*-value = 0.01). The variation in the predicted sampling time was larger during the first 24 hours than in the subsequent 48 hours, which suggests that most of the chromatin remodeling occurs during the first hours after SOX10 knockdown.

Altogether, these results show the utility of ConDecon for estimating cell abundances in bulk tissues profiled with other omics data modalities such as spot-based spatial transcriptomics and ATAC-seq data.

## Discussion

Estimating cell abundances in bulk tissues has been critical to addressing questions related to cellular heterogeneity using bulk transcriptomic data. Although current methods for gene expression deconvolution provide robust and accurate estimates of cell abundances for discrete cell types, they are limited in their ability to infer changes derived from continuous and dynamic cellular processes such as cell differentiation, immune cell activation, or wound healing. The emergence of single-cell RNA-seq technologies in the past decade has provided new powerful avenues for studying questions of cellular heterogeneity in tissues. However, the scalability and applicability of single-cell RNA-seq remains limited. Here, we presented ConDecon, a conceptually different approach to gene expression deconvolution that can detect fine-resolution changes in cell abundance from bulk tissues using single-cell RNA-seq data as reference. ConDecon conceives the bulk tissue as being generated by a stochastic sampling process where cells from the reference single-cell dataset are sampled with different probabilities, and it infers the probability for each cell in the reference dataset to be present in the bulk tissue. The approach thus requires the reference single-cell dataset to be representative of the cell states that are present in the bulk tissue but not necessarily of their cell abundances.

Our analyses using both real and simulated data demonstrate that, similar to current methods for gene expression deconvolution, ConDecon can accurately estimate cell abundances associated with discrete cell types. However, in contrast to those methods, it can also recapitulate gradual changes in cell state that would otherwise be obscured by conventional clustering-based approaches. We have demonstrated the utility of this type of inference in biomedical applications by reanalyzing published data of pediatric ependymal tumors and have discovered a previously unreported implication of microglial neurodegenerative gene expression programs in the mesenchymal transformation of these tumors. We expect that the development of advanced methods for simulating realistic bulk gene expression data from single-cell RNA-seq data^54^ will improve the predictive power of ConDecon. In addition, we have shown that the approach of ConDecon can be adapted to other omics data modalities, such as spatial transcriptomics and chromatin accessibility data, for which there is currently a scarcity of deconvolution approaches. We anticipate that these features will improve our understanding of cellular heterogeneity and tissue cell composition by greatly facilitating the inference of cell state abundances within complex bulk tissues, particularly in the context of evolving systems like development and disease progression.

## STAR Methods

### Resource availability

#### Lead contact

Further information and requests for resources and reagents should be directed to and will be fulfilled by the lead contact, Pablo G. Camara (pcamara@pennmedicine.upenn.edu).

#### Materials availability

This study did not generate new unique reagents.

#### Data and code availability

- This paper analyzes existing, publicly available data. The accession numbers for the datasets are listed in the key resources table.
- Full, unedited IHC images have been deposited in Mendeley Data. Accession numbers are listed in the key resources table.
- All original code has been deposited at GitHub and is publicly available as of the date of publication. DOIs are listed in the key resources table.
- Any additional information required to reanalyze the data reported in this paper is available from the lead author upon request.

### Method details

#### Overview of ConDecon

ConDecon uses the count matrix and latent space of the reference single-cell RNA-seq dataset to estimate the likelihood for each cell in the dataset to be present in the query bulk tissue sample. For that purpose, it considers the set 𝒢 of genes included in both the single-cell and bulk gene expression datasets and the subset 𝒯⊂𝒢 of most variable genes used to build the single-cell gene expression latent space. For each cell in the single-cell RNA-seq dataset, ConDecon aggregates the gene expression counts of the cell and its *r* nearest neighbors in the latent space (default *r* = 5) and computes the Pearson’s correlation between the ranks of 𝒯 in 𝒢 when the elements of 𝒢 are ordered according to the expression values in the bulk and in the aggregated cells. We denote by $\vec{c}$ the vector of correlation coefficients computed in this manner across all cells in the single-cell dataset.

The goal of ConDecon is to infer a vector $\vec{p}$ of cell probabilities starting from $\vec{c}$. For that purpose, it is convenient to expand $\vec{c}$ and $\vec{p}$ in an orthonormal basis of functions with support on the latent space of the single cell dataset,

(1) $$\vec{x}=Z^{-1}\vec{c},\;\vec{y}=Z^{-1}\vec{p}.$$

In these expressions, ***Z*** is a *J* by *D* matrix containing the cell loadings in the latent space and $\vec{x}$ and $\vec{y}$ are vectors in *D* dimensions, where *J* is the number of cells in the single-cell dataset and *D* is the number of dimensions of the latent space (default *D* = 10). By reducing the dimensionality of the problem in this manner, we facilitate learning the relationship between $\vec{p}$ and $\vec{c}$,

(3) $$\vec{p}=Z\vec{h}(\vec{x}).$$

ConDecon uses a polynomial model of degree *s* for $\vec{h}(\vec{x})$ (default *s* = 1),

(3) $$h_{i}(\vec{x})=\beta_{0,i}+\sum_{j=1}^{D}\beta_{1,ij}x_{j}+\sum_{k=1}^{D}\sum_{j=1}^{D}\beta_{1,ij}x_{j}x_{k}+\cdots .$$

To estimate the coefficients *β*, it generates a training dataset consisting of *k* simulated cell abundance distributions in the latent space (default *k* = 5,000), ${\vec{p}}^{\left(l\right)}$, *l* = 1, …, *k*, where each cell abundance distribution is modeled as a mixture of *M*^(*l*)^ *D*-dimensional Gaussian distributions,

$${\vec{p}}^{\left(l\right)}=\sum_{m=1}^{M^{\left(l\right)}}w_{m}^{\left(l\right)}𝒩\left(\vec{z}|{\text{μ}}_{m}^{\left(l\right)},\;\sigma_{m}^{\left(l\right)}\right).$$

The number of components, *M*^(*l*)^, is uniformly sampled from a range of values (default *M*^*(l)*^ ∈ [1,5]); the location of each center ${\text{μ}}_{m}^{\left(l\right)}$ in the latent space is given by a randomly sampled cell from the single-cell data; the covariant matrix $\sigma_{m}^{\left(l\right)}$ is taken to be proportional to the identity with proportionality constant uniformly sampled from a finite range of values such that the fraction of cells within two standard deviations of the center is in a given percentile range (default 5–20%); and each mixing parameter $w_{m}^{\left(l\right)}$ is uniformly sampled from [0,1]. For each probability distribution ${\vec{p}}^{\left(l\right)}$, *n* cells are sampled (with replacement) from the single-cell data (default $n=⎣\frac{j}{2}⎤$), their gene expression counts are aggregated to create a synthetic bulk gene expression profile, and a vector of correlation coefficients ${\vec{c}}^{\left(l\right)}$ is computed as described above. The pairs (${\vec{p}}^{\left(l\right)}$, ${\vec{c}}^{\left(l\right)}$) are then transformed into pairs (${\vec{y}}^{\left(l\right)}$, ${\vec{x}}^{\left(l\right)}$) using equations (1), and they are used to estimate the coefficients *β* in equation (3) using maximum likelihood inference.

The final vector $\vec{p}$ inferred by ConDecon (equation (2)) is then normalized as,

$${\vec{p}}^{\prime }=\frac{\vec{p}}{\|\vec{p}{\|}_{1}}$$

where $\|\vec{p}{\|}_{1}$ denotes the L^1^-norm of $\vec{p}$.

#### Deconvolution of simulated gene expression data

We used the R package splatter^26^ (v1.10.1) to simulate single-cell RNA-seq data with either a distinct number of cell types (splatSimulateGroups) or a continuous cell differentiation trajectory (splatSimulatePaths). Each simulation contained 5,000 cells (batchCells), 20,000 genes (nGenes), approximately 45 positively differentially expressed genes per group, and approximately 5 negatively differentially expressed genes per group (de.prob = 0.0025, de.downprob = 0.1).

##### Simulation of discrete cell types.

To simulate discrete cell types, we generated synthetic single-cell data containing either 3, 6, or 9 cell types of equal size (group.prob) and 9 levels of differentially expressed genes (de.facLoc ∈ [0.01, 0.05, 0.1, 0.15, 0.2, 0.3, 0.4, 0.5, 0.6]). For each of the 27 simulated single-cell datasets, we generated 25 corresponding bulk gene expression profiles by aggregating cells from each cell type *k* with varying proportions $\vec{f}$. For that purpose, *n*_*k*_ cells were uniformly sampled (without replacement) from each cell type *k*, such that *f*_*k*_ ∈ [0.05,1], $\sum_{k=1}^{I}f_{k}=1$, and $n_{k}=⎣f_{k}*\frac{5000}{I}⎤$ where *f*_*k*_ is the simulated abundance of cell type *k* and *I* is the total number of cell types. For each of the 27 simulations, we ran ConDecon with default parameters using the top 10 principal components and 2,000 variable genes calculated with the R package scran^55^ (v1.14.6). We aggregated ConDecon’s inferred cell probabilities ${\vec{p}}^{\prime }$ into inferred cell type abundances ${\vec{f}}^{\prime }$,

$$f_{k}^{\prime }=\sum_{i\in C_{k}}p_{i}^{\prime }$$

and compared them with the simulated cell type abundances *f*_*k*_ by computing their Pearson correlation across samples (cell-type-level performance) or cell types (sample-level performance).

##### Simulation of cell differentiation trajectories.

We simulated single-cell data of cell differentiation trajectories with three different topologies (a tree with a bifurcation (path.from = c(0, 1, 1)), a tree with a three-way split (path.from = c(0, 1, 1, 1)), and a tree with two consecutive bifurcations (path.from = c(0, 1, 1, 3, 3))) and 13 levels of differential gene expression (de.facLoc ∈ [0.01, 0.05, 0.1, 0.15, 0.2, 0.3, 0.4, 0.5, 0.6, 0.7, 0.8, 0.9, 1], such that each branch of the trajectory is approximately straight (path.sigmaFac = 0.5), has genes expressed in a nonlinear manner along the path (path.nonlinearProb = 0.3), and is composed of approximately the same number of cells (group. prob). Pseudotime was interpolated across each trajectory such that there were approximately 20 cells in each iterative step of pseudotime (path.nSteps). For each of the 39 simulated single-cell datasets, we generated 25 bulk gene expression profiles by aggregating 1,000 cells sampled from the single-cell data (with replacement) based on a randomly generated Gaussian distribution *N*(μ, *σ*) where μ is a uniformly sampled cell along pseudotime and σ is uniformly sampled from a range of sigma values that are calculated to on average capture 300 – 1,500 cells within two standard deviations of a center. For each of the 39 simulations, we ran ConDecon with default parameters using the top 10 principal components and 2,000 most variable genes calculated with scran (v1.14.6). To evaluate performance, we calculated the Pearson’s correlation coefficient between ConDecon’s inferred cell probabilities and the simulated ground truth cell abundances.

#### Comparison to clustering-based methods for gene expression deconvolution

We used the benchmarking pipeline of Avila-Cobos *et al.*^27^ to evaluate the ability of ConDecon and 17 other deconvolution methods to infer discrete cell type abundances in bulk tissues. This pipeline builds synthetic bulk RNA-seq datasets by aggregating the gene expression counts of cells sampled from real single-cell RNA-seq datasets. We used 6 single-cell RNA-seq datasets^10,11,28–31^, which we filtered using the same quality control steps outlined in Avila-Cobos *et al.*^27^. In brief, we filtered out genes expressed in less than 5% of the cells, and cells with a total, mitochondrial, or ribosomal UMI count greater than 3 deviations from the median across all the genes. We only considered cell types with at least 50 cells. We down sampled the bone marrow dataset to 8,000 cells. Datasets were then processed using the Seurat pipeline^56^. Cells were log-normalized by library size and the top 2,000 most variable genes were selected for Principal Component Analysis (PCA). For datasets that contained more than one sample (all except for Newman *et al.*^10^), we used Harmony with default parameters to consolidate the top 30 principal components across samples. Cells were clustered using Louvain community detection based on the top 30 latent dimensions and cell populations were annotated using the same sets of markers as in the original papers. As described in Avila-Cobos *et al.*^27^, each single-cell dataset was then subset into two equal-sized datasets representing a reference single-cell dataset and a single-cell dataset that was used to build 1,000 synthetic bulk query datasets. Each synthetic bulk query dataset was constructed by aggregating the gene expression counts of *n* cells sampled from each cell type with different proportions, where the number of sampled cells depended on the size of the dataset (Oetjen *et al.*^30^, *n* = 3,000; Baron *et al.*^11^, *n* = 3,500; Enge *et al.*^28^, *n* = 1,000; Han *et al.*^31^, *n* = 3,000; Segerstolpe *et al.*^29^, *n* = 400; Newman *et al.*^10^, *n* = 3,500). For each dataset, either none, the smallest, medium, or largest cell type was removed from the reference single-cell data to evaluate the stability of the estimates against missing data. The largest, medium, and smallest cell type were defined as the cell population with respectively the largest, median, and smallest number of cells in the reference dataset. We quantified the performance of each algorithm across each dataset and condition by computing the average Pearson’s correlation coefficient and root mean squared error of the predicted values in comparison with the simulated ground-truth values, combined across all samples and cell types. In these analyses, we ran ConDecon with default parameters using the top 10 latent dimensions and 2,000 most variable genes computed with Seurat (v3.1.5). We aggregated ConDecon’s inferred cell probabilities into inferred cell type proportions as described above. All the other gene expression deconvolution methods were run with default parameters using all the marker genes when applicable^27^.

#### Comparison of estimated cell type abundances to FACS data

We benchmarked ConDecon, CIBERSORTx^10^ (S-mode), MuSiC^12^, Bisque^32^, and CPM^33^ using two bulk RNA-seq datasets of human bone marrow^30^ and PBMC^10^ for which paired FACS data were available. In these analyses, ConDecon and all the other algorithms were run with default parameters. CIBERSORTx, MuSiC, and Bisque were used with the marker genes outputted by the “Create Signature Matrix” tool of CIBERSORTx. CPM was run using quantifyTypes = TRUE and a homogeneous 2-dimensional representation, as recommended in its documentation (https://github.com/amitfrish/scBio). To build the homogeneous 2-dimensional representation, we applied UMAP to the top 30 principal components based on the top 2,000 least variable genes expressed in > 5% of cells. For each bulk sample, the cell weights inferred by CPM were shifted by their minimum value so that they were all positive, and then normalized as probabilities and aggregated into cell type proportions. To evaluate the performance of each algorithm, we calculated the Pearson’s correlation coefficient between the predicted cell type proportions and the FACS data across bulk samples (cell-type-level performance) or cell types (sample-level performance).

#### Analysis of B cell maturation

We considered the longitudinal mouse bone marrow single-cell RNA-seq dataset from the Tabula Muris Consortium^34^, which consists of 13 and 54 mice profiled at the single-cell and bulk level, respectively. We removed one bulk sample that had < 1,000 expressed genes from downstream analyses. We followed the same quality control procedures described above (subheading “Comparison to clustering-based methods for gene expression deconvolution”) to process the single-cell RNA-seq data, with the addition of removing genes expressed in < 0.1% of the cells. We used Seurat^56^ (v3.1.5) to log-normalize the gene expression profile of each cell by library size and perform PCA using the top 2,000 variable genes. We then consolidated the single-cell data of the 13 mice using Harmony with default parameters and the top 30 principal components. The resulting consolidated latent space was clustered using Louvain community detection and the clusters were annotated using the marker genes identified in the original reference. We used Monocle 3^57^ (v0.2.3) with default parameters to infer a cell differentiation pseudotime $\left(\vec{t}\right)$ in the single-cell gene expression space of the B cell lineage. We then ran ConDecon, CIBERSORTx (S-mode), MuSiC, Bisque, and CPM as described above (subheading “Comparison of estimated cell type abundances to FACS data”). We evaluated the performance of these methods by calculating the log_2_-fold change of the median predicted cell type proportion of each B cell subpopulation between samples from young (≤ 3 months) and adult (> 3 months) mice. Statistical significance was calculated using a Wilcoxon rank sum test. In addition, we used the single-cell probabilities ${\vec{p}}^{\prime }$ inferred by ConDecon to compute the estimated average pseudotime of the B cells in each bulk sample, $E[t]=\left({\vec{p}}^{\prime }\cdot \vec{t}\right)/\|\vec{p}{\|}_{1}$, where only the interquartile range of the distribution of probabilities was considered in the estimation. We then tested the association between the estimated average pseudotime and the mouse age of each sample using Pearson’s correlation.

#### Gene expression deconvolution of ependymoma RNA-seq data

We downloaded the processed bulk (42 tumors) and single-nucleus RNA-seq data of pediatric ependymal tumors from Aubin *et al*.^21^ and applied ConDecon with default parameters using the top 5 latent dimensions and 5,000 variable genes. We assigned a score to each tumor cell in the single-nucleus data to represent its stage in the neuroepithelial-like to mesenchymal-like cell state transition. For that purpose, we aggregated for each cell the normalized expression values of the genes that are differentially expressed (FDR < 0.05, log fold-change > 3) between the clusters of neuroepithelial- and mesenchymal-like tumor cells. Similarly, we assigned a score representing the transition from a basal into a DAM state to each microglia in the single-nucleus data by aggregating the normalized expression values of the genes belonging to the DAM gene expression signature of Butovsky and Weiner^24^ (c.f. Fig. 2a in that reference). We then estimated the average scores of the tumor and microglial cells in each bulk sample using the same approach described above based on the inferred probabilities ${\vec{p}}^{\prime }$ of ConDecon (subheading “Analysis of B cell maturation”). We tested the association between the estimated average microglial and mesenchymal scores of each bulk sample using Pearson’s correlation.

#### Application of ConDecon to spatial transcriptomics data

We applied ConDecon to published Stereo-seq and single-cell RNA-seq data of the zebrafish embryo from Liu *et al*.^25^. The normalized gene expression data and cell type annotations were downloaded via h5ad files provided by the paper for the single-cell RNA-seq and Stereo-seq datasets of 3.3 hpf embryos. We filtered out genes expressed in < 3 cells, log-normalized gene counts, and identified variable genes using Scanpy^58^ (v1.9.1) (scanpy.pp.highly_variable_genes() function with default parameters). We used the top 3,350 variable genes to perform PCA and identified clusters with the Leiden algorithm at a resolution of 0.5 using the top 20 principal components. We computed diffusion pseudotime^45^ for the single-cell RNA-seq data using the scanpy.tl.dpt() function with default parameters, setting the cluster with highest expression of early blastodisc markers (otx1, dvl2, ctnnb1, axin1)^25^ as the root for the pseudotime calculation. We applied ConDecon with default parameters to infer single-cell abundances for each Stereo-seq pixel. We estimated the average pseudotime of each pixel using the approach described in subheading “Analysis of B-cell maturation”. To visualize the spatial cell differentiation trajectories derived from the inferred pseudotime spatial patterns, we computed the gradient over the pseudotime of each pixel using the immediately adjacent pixels. The resulting gradient vectors were smoothed using a gaussian kernel with standard deviation of 30 μm, truncated at 40 μm. We visualized the smoothed vector field using the matplotlib.pyplot.streamplot() function of Matplotlib (v3.5.3) with density = 1.5.

#### Application of ConDecon to ATAC-seq data

We applied ConDecon to published bulk and single-nucleus ATAC-seq data from two patient-derived melanoma cell lines (MM057 and MM087) profiled 0, 24, 48, or 72 hours after knocking down SOX10^53^. We used the 288 cells from MM087 as single-nucleus ATAC-seq reference data. To create a set of common peaks between the bulk and single-nucleus ATAC-seq data, we binned the genome into non-overlapping 10 kilobase bins. For each bin, we then aggregated the peaks that overlapped the bin. We assigned peaks that overlapped more than one bin to the bin with the smallest genome coordinates among the two overlapping bins. This resulted in 24,234 and 53,833 accessible bins in the single-nucleus and bulk data, respectively. To build a single-nucleus ATAC-seq data latent space, we used latent semantic indexing (LSI) as implemented in Signac^59^ (v1.1.0). We used bins that were open in at least 90% of the cells for term frequency-inverse document frequency (TF-IDF) normalization followed by singular value decomposition (SVD). We neglected the first component and visualized components 2 to 20 in two dimensions using UMAP. We applied ConDecon (with parameters max.center = 1, sigma_min_cells = 30, and sigma_max_cells = 75) to the bulk and single-nucleus bin-count matrices, using dimensions 2 to 11 of the latent space and the top 90% most variable bins (n = 22,362). We then estimated the average sampling time of the cells in each bulk sample based on the single-cell probabilities ${\vec{p}}^{\prime }$ inferred by ConDecon (see subheading “Analysis of B-cell maturation”) and tested the association between the estimated average sampling time of the cells and the actual sampling time using Pearson’s correlation.

#### Immunohistochemistry of ependymal tumors

De-identified formalin-fixed paraffin-embedded (FFPE) 5 μm tissue sections from two primary (7316-496 and 7316-509) and one metastatic (7316-490, cortical metastasis) ependymal tumors located in or derived from the posterior fossa were provided by the Children’s Brain Tumor Tissue Network (CBTN) biorepository (Approved Biospecimen Project #29). The anatomic location of the tumors and their diagnosis were obtained from the surgical, radiology, and pathology reports. All the tissue sections and data were provided by the CBTN in a deidentified form according to the U.S. Department of Health and Human Services regulations and were not considered as Human Subjects Research by the Institutional Review Board of the University of Pennsylvania. Tissue handling procedures were performed according to the institutional regulations of the University of Pennsylvania and the Childreńs Hospital of Philadelphia (CHOP). Adjacent FFPE sections from each of the 3 tumors were stained with anti-CA9 (Novus, NB100-417SS), anti-IBA1 (Wako, 019-19741), and anti-GPNMB (R&D Systems, AF2550). Staining was performed on a Bond Max automated staining system (Leica Biosystems). The Bond Refine polymer staining kit (Leica Biosystems DS9800) was used for anti-IBA1 and anti-CA9. The Intense-R staining kit (Leica Biosystems, DS9263) was used for anti-GPNMB. The standard protocols were followed except for the primary antibody incubation which was extended to 1 hour at room temperature. Antibodies were used at the following dilutions: anti-IBA1 1:2,000, anti-CA9 1:1,000, anti-GPNMB 1:500. Antigen retrieval was performed with E2 (anti-IBA1) or E1 (anti-CA9, anti-GPNMB) (Leica Microsystems) retrieval solution for 20 min. Slides were rinsed, dehydrated thru a series of ascending concentrations of ethanol and xylene, then cover-slipped. Stained slides were then digitally scanned at 20x magnification on an Aperio AT2 slide scanner (Leica Biosystems).

### Quantification and statistical analysis

Two-sided Pearson correlation test of associations were used in Figures 2B, 2D, 3A, 3B, 4C, 5D, 6G, S1, S2B, S3, and S4. Two-sided Wilcoxon rank-sum tests were used in Figures 4E and 6D. P values and sample sizes for each statistical test are described in the respective figure legend.

## Supplementary Material

Supplement 1

Supplement 2
