# Supplementary material for: Clustering-independent estimation of cell abundances in bulk tissues using single-cell RNA-seq data
Source: bioRxiv. 2023 Feb 7:2023.02.06.527318. Preprint. [Version 1] doi: 10.1101/2023.02.06.527318 (PMC9934539; doi:10.1101/2023.02.06.527318)
Supplement: Supplementary file 1 [file media-1.pdf]

Supplemental Figures

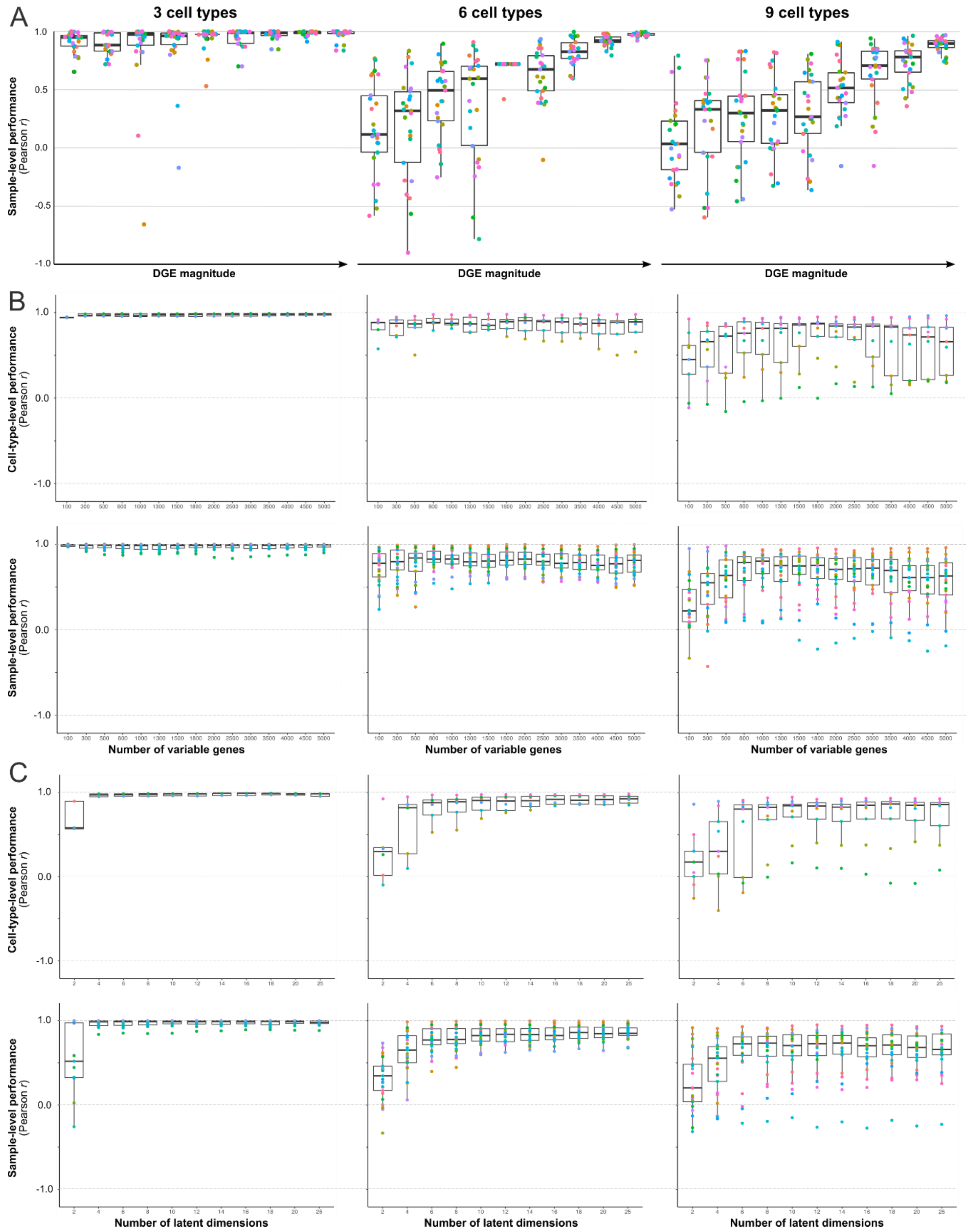

Figure S1. Deconvolution of simulated bulk RNA-seq data of discrete cell populations. (A)

Sample-level Pearson correlation coefficient between simulated and estimated cell population

abundances in simulations of bulk RNA-seq datasets (*n* = 675) with 3, 6, or 9 discrete cell populations and varying degree of differential gene expression (DGE). **(B, C)** Cell-type- and sample-level Pearson correlation coefficient between simulated and estimated cell population abundances in simulated bulk RNA-seq datasets (*n* = 675) with 3, 6, or 9 discrete cell populations as a function of the number of variable genes (B) and latent dimensions (C) used by ConDecon.

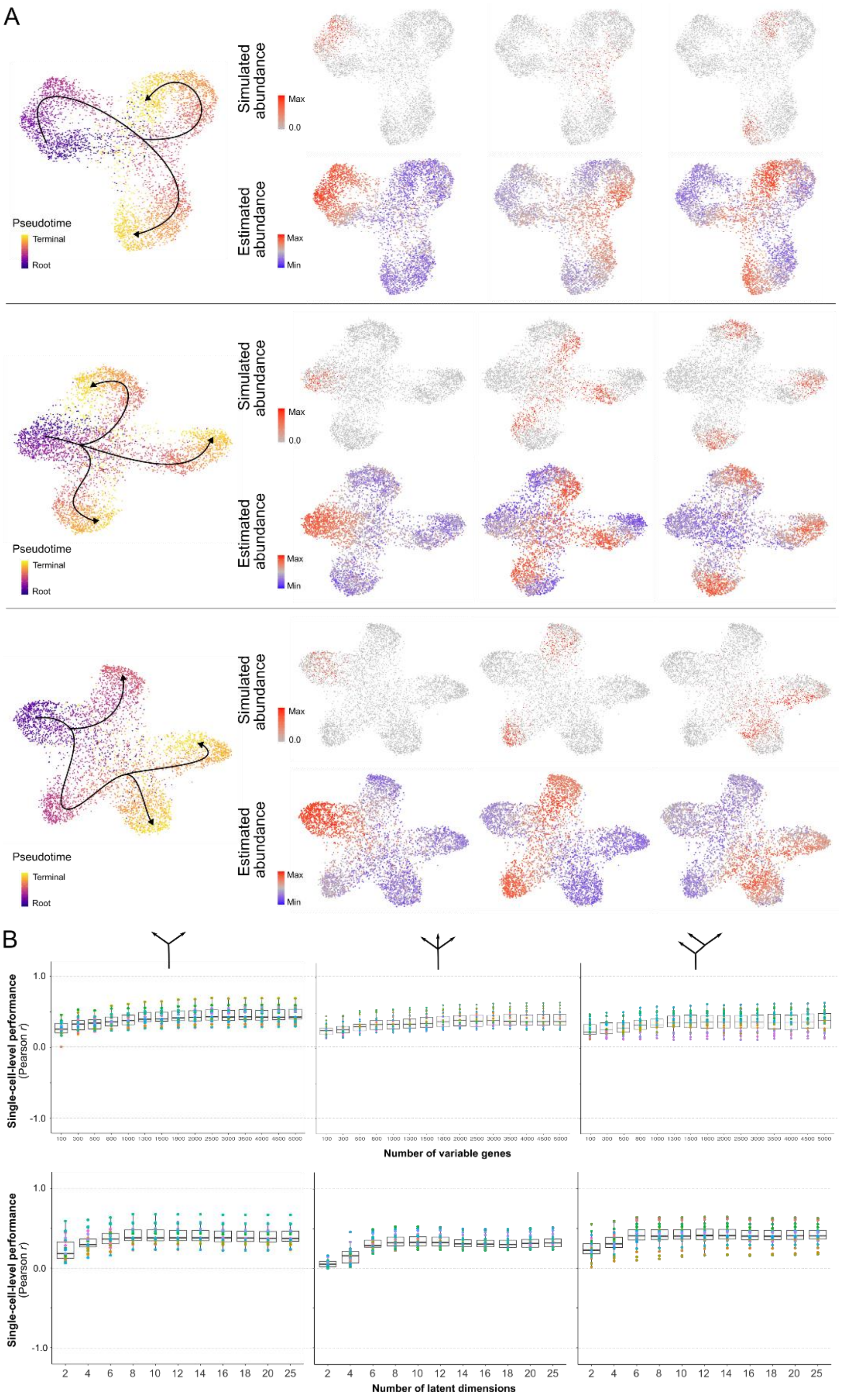

Figure S2. Deconvolution of simulated bulk RNA-seq data of cell differentiation processes.

(A) Cell abundance estimation in 9 simulated bulk RNA-seq datasets of 3 cell differentiation

processes with 1 precursor and 2 or 3 terminally differentiated cell states. Left: The UMAP representation of each simulated single-cell RNA-seq dataset is colored by the simulated pseudotime. Right: For each single-cell dataset, the simulated (top) and estimated (bottom) cell abundances are shown for 3 bulk RNA-seq datasets constructed by sampling cells non-uniformly from the single-cell dataset. **(B)** Single-cell-level Pearson correlation coefficient between simulated and estimated cell abundances in simulations of bulk RNA-seq datasets with 3 different topologies as a function of the number of variable genes (top) and latent dimensions (bottom) used by ConDecon. The topologies of the cell differentiation processes are indicated at the top.

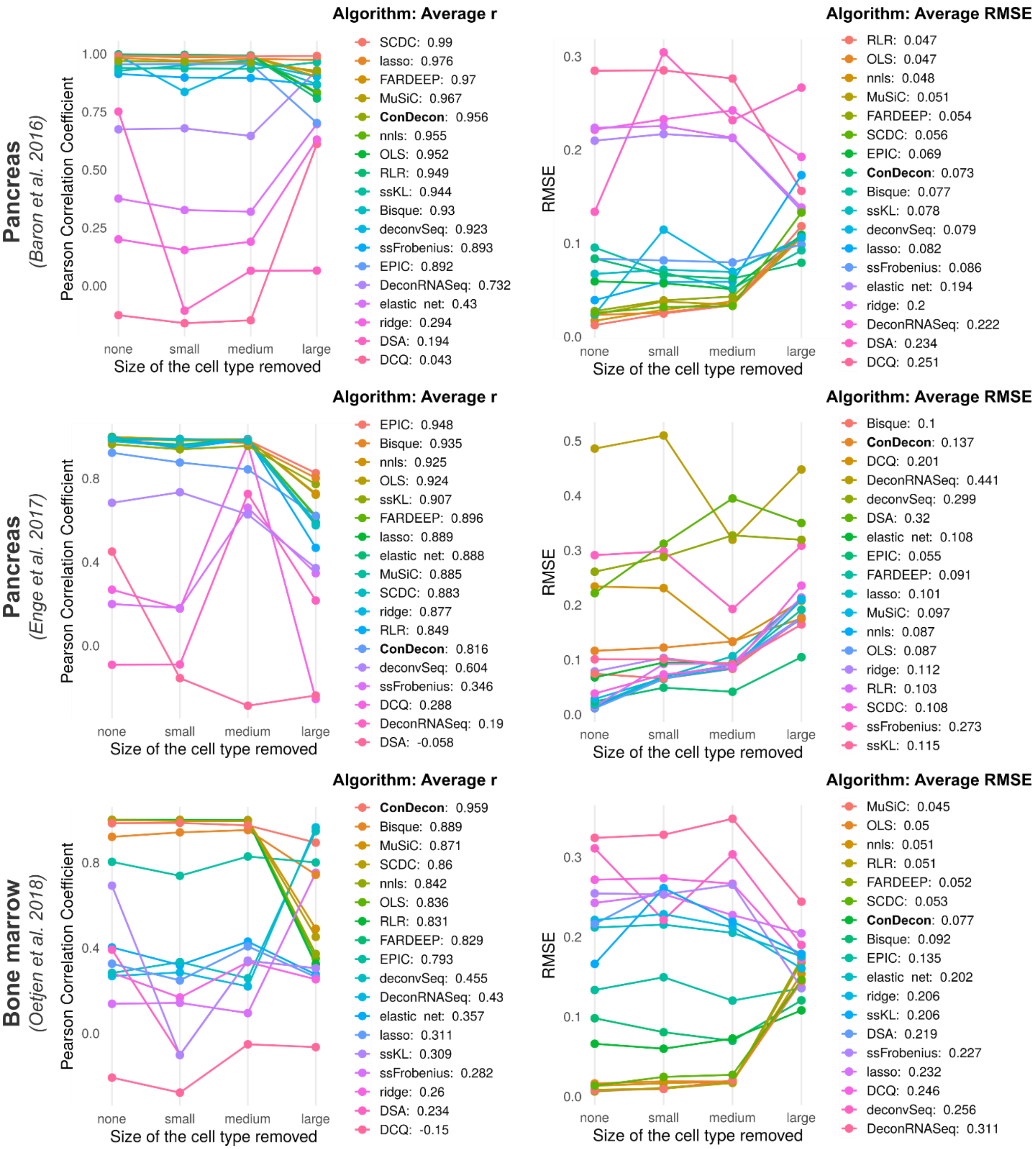

(cont. on next page)

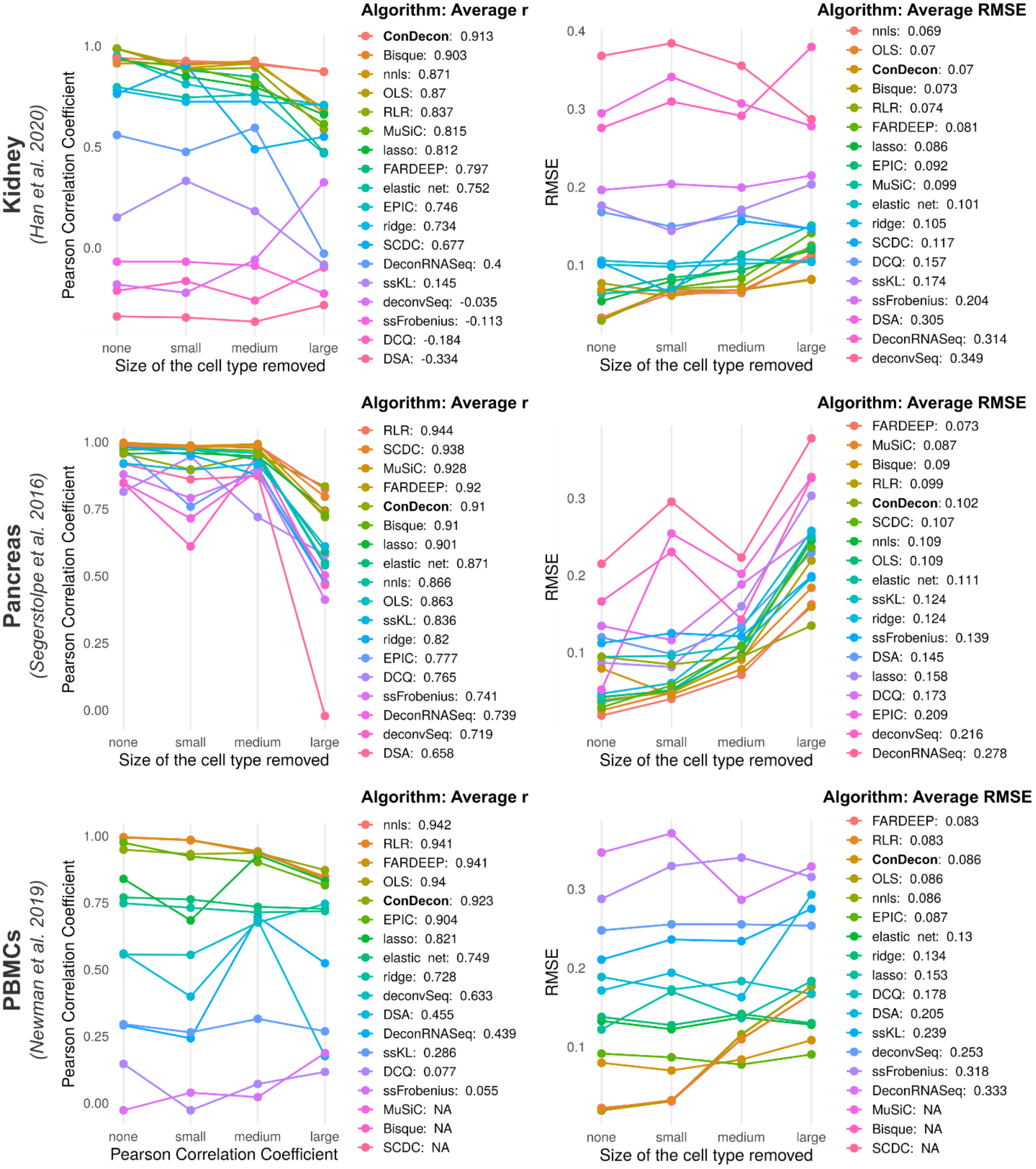

Figure S3. Benchmarking of the aggregated cell abundance estimates of ConDecon in comparison to seventeen other methods for gene expression deconvolution. Evaluation of the aggregated cell abundance estimates of ConDecon and the cell type abundance estimates of 17 other deconvolution methods across 6 datasets using the benchmarking pipeline of Avila-

Cobos *et al.* For each algorithm and dataset, the Pearson’s correlation coefficient (left) and the root mean squared error (RMSE) (right) of the estimates, combined across samples and cell types, is shown for cases where there is none, one small, one medium, or one large cell population missing in the reference single-cell data.

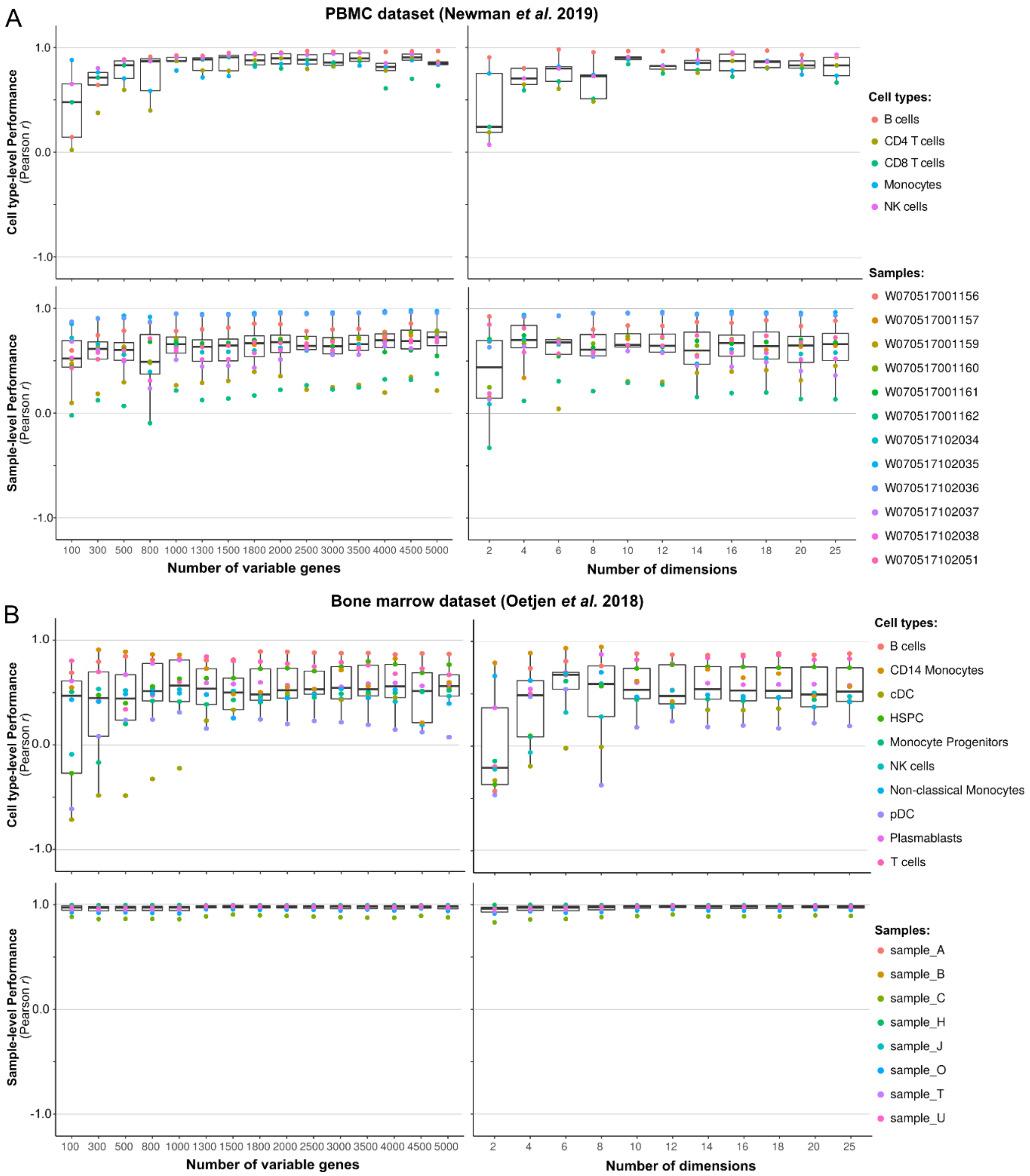

Figure S4. Deconvolution of bulk RNA-seq data of PBMCs and bone marrow as a function of ConDecon's parameters. Single-cell-level Pearson correlation coefficient between the estimated cell abundances by ConDecon and the observed abundances by FACS for bulk RNA-seq datasets consisting of 8 bone marrow[1] (A) and 12 PBMC[2] (B) samples as a function of the number of variable genes (top) and latent dimensions (bottom) used by ConDecon.

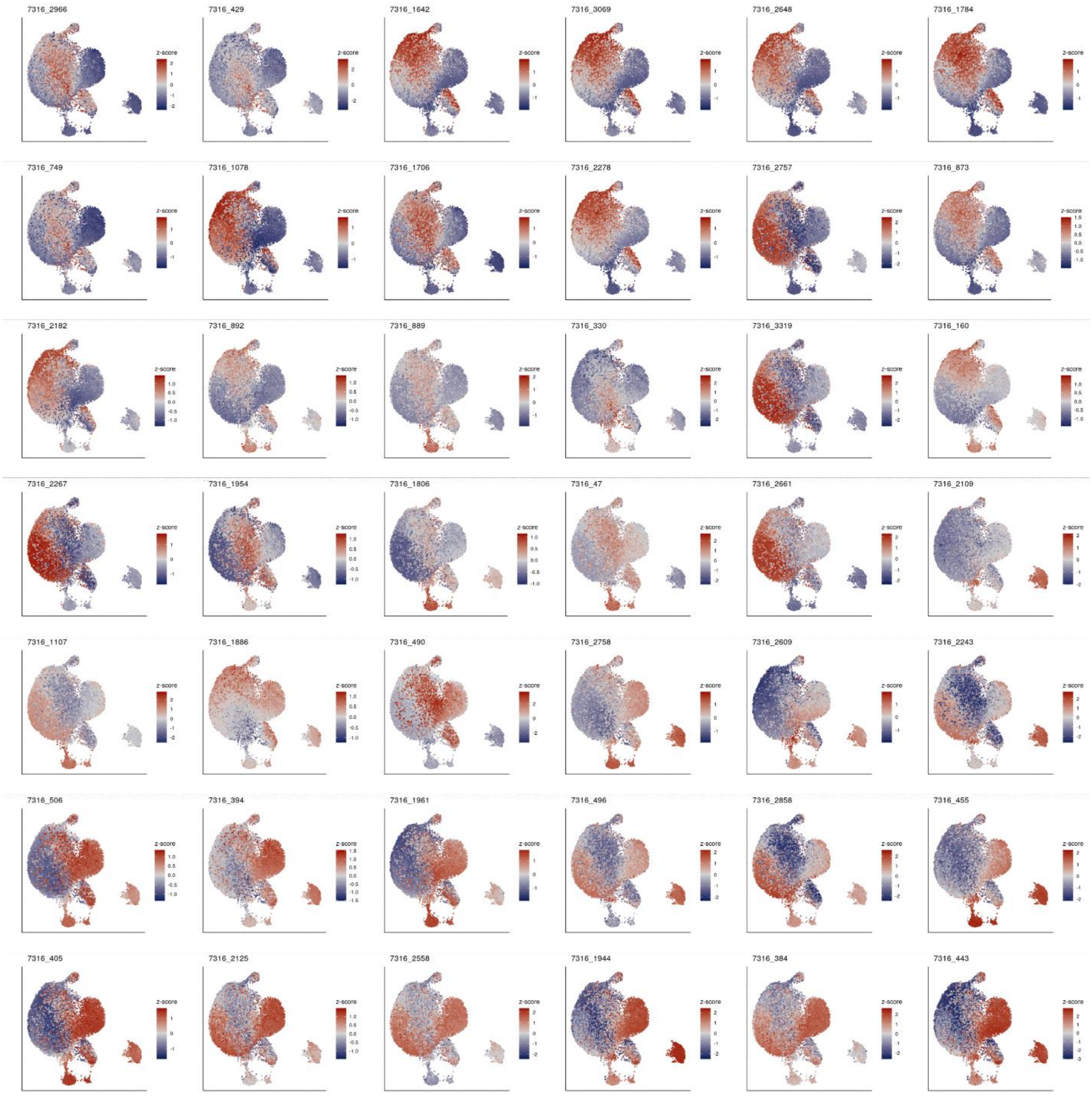

Figure S5. Deconvolution of bulk RNA-seq data of pediatric ependymoma. Single-cell abundance estimates computed with ConDecon for 42 pediatric ependymal tumors in the posterior fossa profiled with bulk RNA-seq. The UMAP representation of 25,349 cells from 9 posterior fossa ependymal tumors profiled with single-nucleus RNA-seq in Aubin *et al.*[3] is colored by the inferred single-cell abundances. Tumors are arranged from left to right and top to bottom according to their inferred state in the neuroepithelial-to-mesenchymal transition.

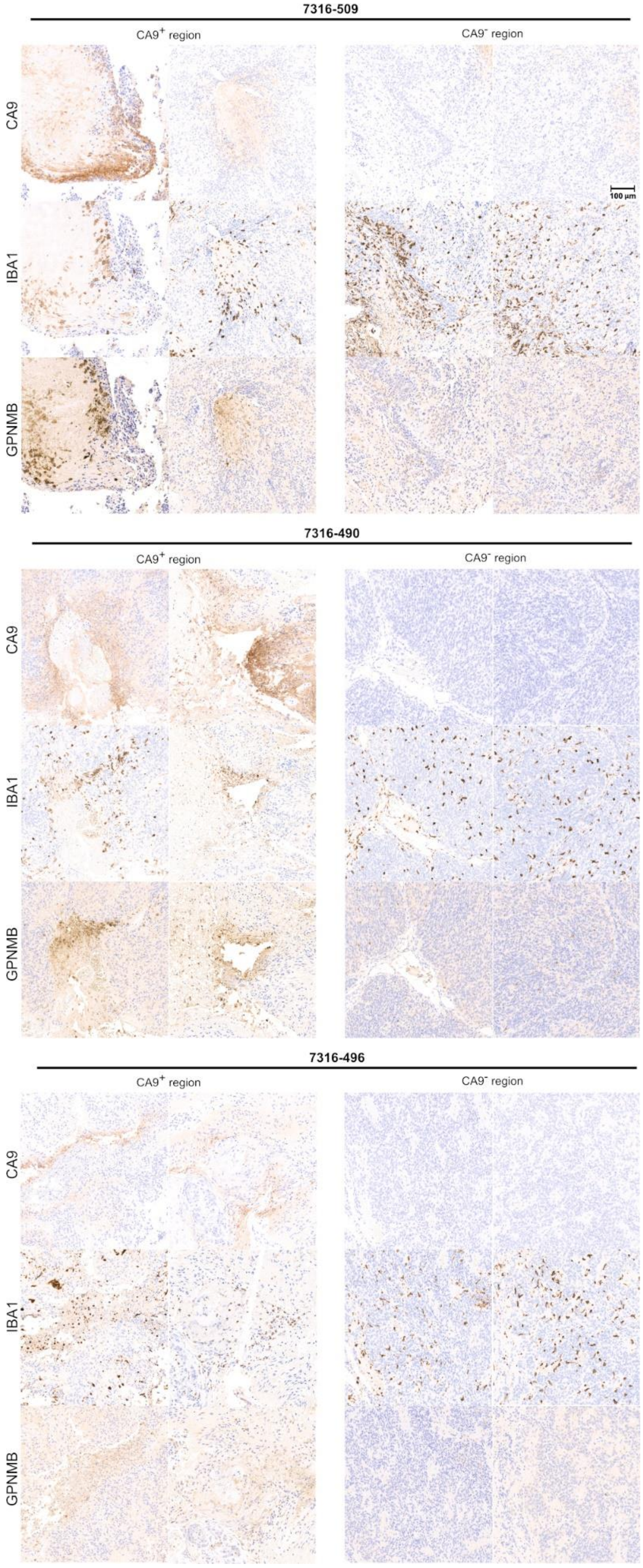
7316-509
CA9+ region
CA9- region
CA9
IBA1
GPNMB
100 µm
7316-490
CA9+ region
CA9- region
CA9
IBA1
GPNMB
7316-496
CA9+ region
CA9- region
CA9
IBA1
GPNMB

Figure S6. Immunohistochemistry staining of adjacent tissue sections from pediatric ependymal tumors. Two primary (7316-509 and 7316-496) and one metastatic (7316-490). Each tumor was stained for CA9 (a marker of mesenchymal regions), IBA1 (a microglial marker), and GPNMB (a DAM marker). In both tumors, microglia that are infiltrating $CA9^{+}$ mesenchymal regions express GPNMB, whereas microglia in neuroepithelial regions of the tumor do not have detectable levels of GPNMB. Scale bar: 100 μm.

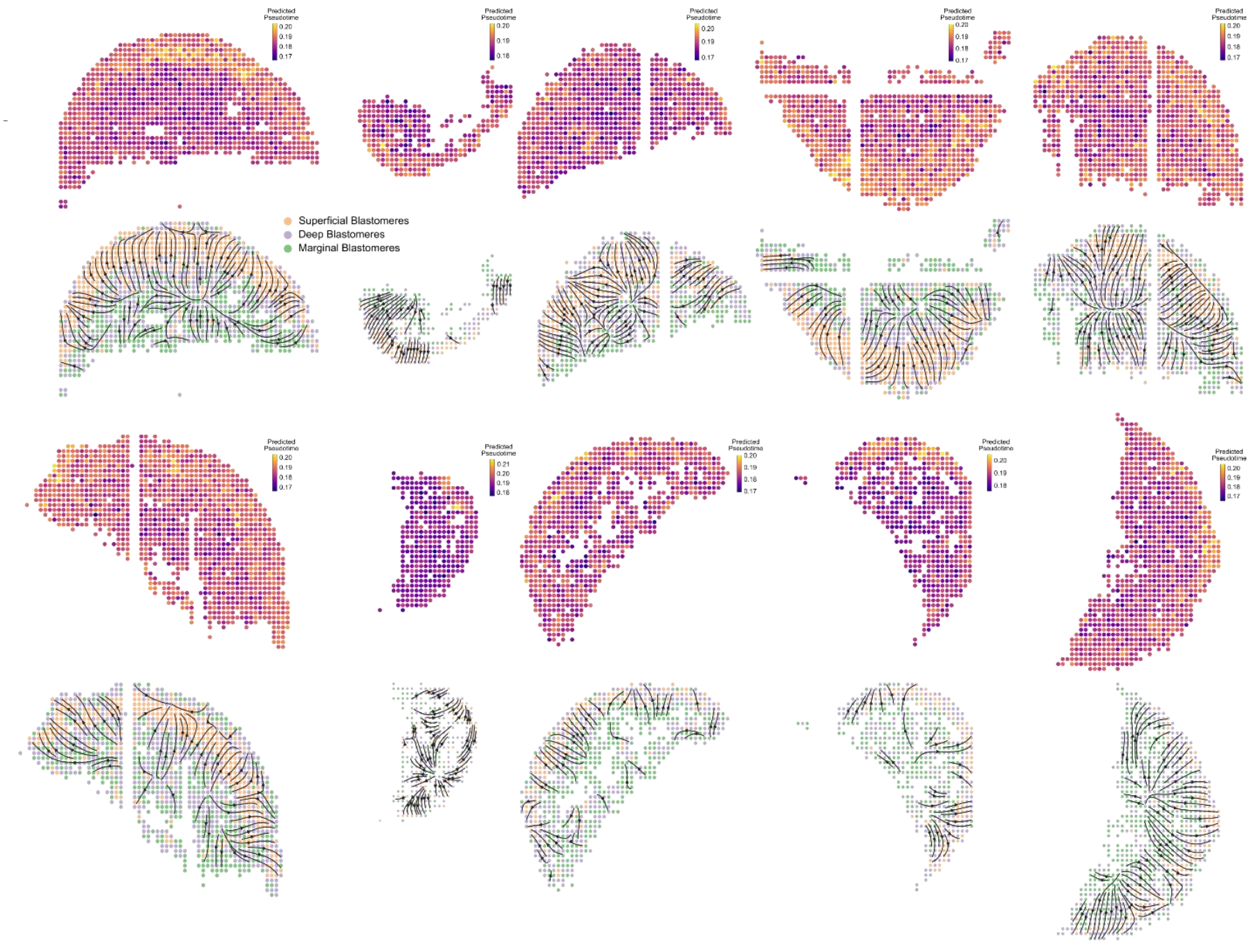

Figure S7. Deconvolution of spatial transcriptomic data of zebrafish embryos. Spatial tissue sections of 10 3.3 hpf zebrafish embryos profiled with Stereo-seq[4]. Each section is labeled by the average pseudotime estimated with ConDecon for the cells in each pixel (top) and the corresponding spatial cell differentiation trajectories (bottom).

References

1. Oetjen, K.A., Lindblad, K.E., Goswami, M., Gui, G., Dagur, P.K., Lai, C., Dillon, L.W., McCoy, J.P., and Hourigan, C.S. (2018). Human bone marrow assessment by single-cell RNA sequencing, mass cytometry, and flow cytometry. JCI Insight *3*. 10.1172/jci.insight.124928.
2. Newman, A.M., Steen, C.B., Liu, C.L., Gentles, A.J., Chaudhuri, A.A., Scherer, F., Khodadoust, M.S., Esfahani, M.S., Luca, B.A., Steiner, D., et al. (2019). Determining cell type abundance and expression from bulk tissues with digital cytometry. Nat Biotechnol *37*, 773-782. 10.1038/s41587-019-0114-2.
3. Aubin, R.G., Troisi, E.C., Montelongo, J., Alghalith, A.N., Nasrallah, M.P., Santi, M., and Camara, P.G. (2022). Pro-inflammatory cytokines mediate the epithelial-to-mesenchymal-like transition of pediatric posterior fossa ependymoma. Nature communications *13*, 3936. 10.1038/s41467-022-31683-9.
4. Liu, C., Li, R., Li, Y., Lin, X., Zhao, K., Liu, Q., Wang, S., Yang, X., Shi, X., Ma, Y., et al. (2022). Spatiotemporal mapping of gene expression landscapes and developmental trajectories during zebrafish embryogenesis. Dev Cell *57*, 1284-1298 e1285. 10.1016/j.devcel.2022.04.009.
