# Supplementary material for: Clustering-independent estimation of cell abundances in bulk tissues using single-cell RNA-seq data
Source: bioRxiv. 2023 Feb 7:2023.02.06.527318. Preprint. [Version 1] doi: 10.1101/2023.02.06.527318 (PMC9934539; doi:10.1101/2023.02.06.527318)
Supplement: Supplementary file 2 [file media-2.pdf]

KEY RESOURCES TABLE

REAGENT or RESOURCE	SOURCE	IDENTIFIER
Antibodies		
Anti-Human Carbonic Anhydrase IX	Novus	Cat# NB100-417SS; RRID:AB_788423
Anti-Human Osteoactivin/GPNMB	R&D Systems	Cat# AF2550; RRID:AB_416615
Anti-Human IBA1	Wako	Cat# 019-19741; RRID:AB_839504
Biological Samples		
PFA Ependymoma FFPE tissue sections	Children's Brain Tumor Network (CBTN)	7316-509
PFA Ependymoma FFPE tissue sections	Children's Brain Tumor Network (CBTN)	7316-490
PFA Ependymoma FFPE tissue sections	Children's Brain Tumor Network (CBTN)	7316-496
Deposited Data		
Single-cell RNA-seq data of human pancreatic islands	Enge et al., 2017	GEO: GSE81547
Single-cell RNA-seq data of human pancreatic islands	Baron et al., 2016	GEO: GSE84133
Single-cell RNA-seq data of human pancreatic islands	Segerstolpe et al., 2016	ArrayExpress: E-MTAB-5061
Single-cell RNA-seq data of PBMC	10x Genomics	https://support.10xgenomics.com/single-cell-gene-expression/datasets/2.1.0/pbmc8k
Single-cell and bulk RNA-seq and FACS data of human bone marrow cells	Oetjen et al., 2018	GEO: GSE120446
Single-cell RNA-seq data of human kidney	Han et al., 2020	https://figshare.com/articles/HCL_DGE_Data/7235471
Bulk RNA-seq and FACS data of PBMC	Newman et al., 2019	GEO: GSE127813; http://cibersortx.stanford.edu
Single-cell RNA-seq data from Tabula Muris Senis	Tabula Muris Consortium et al., 2020	GEO: GSE149590
Bulk RNA-seq data from Tabula Muris Senis	Tabula Muris Consortium et al., 2020	GEO: GSE132040
Single-nucleus RNA-seq data of ependymal tumors	Aubin et al., 2022	GEO: GSE206578
Bulk RNA-seq data of	Aubin et al.,	https://portal.kidsfirstdrc.org, project PBTA-CBTN

ependymal tumors	2022	
Single-cell RNA-seq and Stereo-seq data of zebrafish embryos	Liu et al., 2022	https://db.cngb.org/stomics/zesta/
Single-nucleus and bulk ATAC-seq data of melanoma cell lines	Bravo Gonzalez-Blas et al., 2019	GEO: GSE114557
Software and Algorithms		
ConDecon (v1.0.0)	This paper	https://github.com/CamaraLab/ConDecon
Splatter (v1.10.1)	Zappia et al., 2017	https://github.com/Oshlack/splatter
scran (v1.14.6)	Lun et al., 2016	https://github.com/MarioniLab/scran
Gene expression deconvolution benchmarking pipeline	Avila-Cobos et al., 2020	https://github.com/favilaco/deconv_benchmark
Seurat (v3.1.5)	Satija et al., 2015	https://github.com/satijalab/seurat/tree/v3.1.5
CIBERSORTx	Newman et al., 2019	https://cibersortx.stanford.edu/
MuSiC (v0.2.0)	Wang et al., 2019	https://github.com/xuranw/MuSiC
Bisque (v1.0.5)	Jew et al., 2020	https://github.com/cozygene/bisque
CPM (v 0.1.5)	Frishberg et al., 2019	https://github.com/amitfrish/scBio
Monocle 3 (v0.2.3)	Cao et al., 2019	https://github.com/cole-trapnell-lab/monocle3/tree/0.2.3
Scanpy (v1.9.1)	Wolf et al., 2018	https://github.com/scverse/scanpy/tree/1.9.1
Signac (v1.1.0)	Stuart et al., 2021	https://github.com/stuart-lab/signac/tree/1.1.0
